# Comparison of Traditional Chinese Medicine in the Long-Term Secondary Prevention for Patients with Ischemic Stroke: A Systematical Analysis

**DOI:** 10.3389/fphar.2021.722975

**Published:** 2021-11-18

**Authors:** Jiali Li, Xixi Zhao, Yangyang Zhang, Haitong Wan, Yu He, Xiaohong Li, Li Yu, Weifeng Jin

**Affiliations:** ^1^ The Second School of Clinical Medicine, Zhejiang Chinese Medical University, Hangzhou, China; ^2^ School of Life Sciences, Zhejiang Chinese Medical University, Hangzhou, China; ^3^ School of Pharmaceutical Sciences, Zhejiang Chinese Medical University, Hangzhou, China

**Keywords:** traditional Chinese medicine, ischemic stroke, long-term, secondary prevention, recurrent stroke, stroke risk factors, systematical analysis

## Abstract

**Background:** Keeping in view the high recurrence rate and risk of ischemic stroke, combinatorial therapy involving traditional Chinese medicine (TCM) with conventional Western medicine (WM) is receiving wider scientific attention. Thus, a systematical analysis was made to explore the efficacy of TCM+WM in the long-term secondary prevention for patients with ischemic stroke.

**Methods:** Qualified inclusion and exclusion criteria were set up beforehand, and two researchers independently read the articles, extracted data, and evaluated the quality of included articles according to Cochrane Reviewer’s Handbook 5.1 method. For the sake of comprehensive data acquisition, seven databases from the time of their establishment to May 5, 2021, have been searched completely. Additionally, pairwise meta-analysis was made to compare TCM+WM vs. WM, and network meta-analysis was conducted by frequentist random effects models for the comparison of different kinds of TCM+WM via indirect evidence. The primary outcomes defined were recurrent stroke and NIHSS. Secondary outcomes were fibrinogen (Fib) fasting blood glucose (FBG), triglycerides (TG), and total cholesterol (TC). Safety outcomes were outlined as all-cause mortality and adverse events (AEs). Furthermore, Stata16.0 software was used to accomplish the systematical analysis and cluster analysis.

**Results:** In total, 47 qualified randomized controlled trials (RCTs) including 10,732 patients were taken into consideration. Seven traditional Chinese medicines included in the study are Naoxintong capsule (NXT), Tongxinluo capsule (TXL), Buyang Huanwu decoction (BYHW), Naomaitai capsule (NMT), Dengzhan Shengmai capsule (DZSM), Naoshuantong capsule (NST), and Maixuekang capsule (MXK). With respect to their primary outcomes, all kinds of TCM+WM were significantly more effective than WM (e.g., NXT in recurrent stroke (OR=0.54, P<0.01), TXL in NIHSS (WM=−1.4, P<0.01)). Additionally, the outcomes of cluster analysis indicated that MXK+WM and NST+WM had relatively good preventive effects for recurrent stroke, NIHSS, and all-cause mortality. There was no significant difference in the comparisons of AEs; however, this may arise from the lack of sufficient data.

**Conclusion:** According to our systematical analysis, MXK+WM and NST+WM had relatively good secondary prevention effects for patients with ischemic stroke regarding recurrent stroke, NIHSS, and all-cause mortality. Nevertheless, better, high-quality, large-sample randomized clinical trials (RCTs) are required to verify our conclusions in the future.

**Systematic Review Registration:** [https://inplasy.com/inplasy-2021-5-0036/], identifier [INPLASY202150036].

## Introduction

Stroke causes nearly 5% of all disabilities (Collaborators, 2017b) and about 10% of deaths around the world (Collaborators, 2017a). In addition, ischemic stroke accounts for about 70% of the total death caused by stroke (Feigin et al., 2018). Moreover, a good volume of research showed that the cumulative long-term risk of recurrence of stroke is 11.1% at 1 year and 26.4% at 5 years (Mohan et al., 2011). Besides, patients with ischemic stroke are faced with a higher risk of vascular events or death (Kernan et al., 2014; Amarenco et al., 2016). Furthermore, some researchers said that taking secondary prevention on time will decrease the risk of stroke recurrence by about 80% (Hackam and Spence, 2007; Rothwell et al., 2007). Therefore, it is essential to take appropriate measures to avoid the recurrence of stroke.

According to the American Heart Association/American Stroke Association guideline (AHA/ASA), conventional Western medicine for stroke treatment includes hypoglycemic drugs, antihypertensive drugs, antiplatelet drugs, hypolipidemic drugs, etc. (Kernan et al., 2014). However, the combination of clopidogrel and aspirin is not any more effective than either aspirin or clopidogrel monotherapy, and gives rise to a higher risk of bleeding (Diener et al., 2004; Bhatt et al., 2006; Bhatt et al., 2007). Some patients are resistant to antiplatelet drugs (Guo et al., 2019). Moreover, one study has shown that statins for the treatment of stroke can increase the relative risk of new onset diabetes by 9% (Gao, 2015). New treatment methods are thus warranted.

Traditional Chinese medicine (TCM) is attracting increasing attention on account of its exact curative effect and low toxicity (Bu et al., 2020). In addition, in pre-thrombotic conditions, TCM is a multi-link, multi-target which effectively prevents the recurrence of ischemic stroke (Kang and Li, 2012). Furthermore, TCM plays a significant role in the prevention of cerebrovascular disease and recovery of limb function (Zhang et al., 2012).

For example, a randomized controlled trial (RCT) showed that Naoxintong capsule (NXT) had a positive effect on the prevention of recurrence of stroke (Sun, 2020), and another RCT indicated that Tongxinluo capsule (TXL) promoted the recovery of nerve function (Bo et al., 2017). In this study, NXT, TXL, Buyang Huanwu decoction (BYHW), Naomaitai capsule (NMT), Dengzhan Shengmai capsule (DZSM), Naoshuantong capsule (NST), and Maixuekang capsule (MXK) were made a part of the systematical analysis.

At present, there are only a few reviews and meta-analyses on the long-term secondary prevention of cerebral infarction by the combination of Western medicine (WM) and traditional Chinese medicine (TCM). One of the few studies showed that Buyang Huanwu decoction has no statistical significance in reducing the recurrence rate of ischemic stroke (Xie, 2015), possibly because of the lack of any significant literature at that time. Different from traditional pairwise meta-analysis, network meta-analysis (NMA) allows various treatments to be compared and ranked through direct and indirect contrasts to select the best ones (De Laat, 2017). However, comparisons of efficacy across different types of TCM are still inconclusive, causing confusion among physicians and patients.

Therefore, this study is intended to use systematical analysis including pairwise and network meta-analysis to compare the long-term secondary prevention effects and safety of the addition of different kinds of traditional Chinese medicines against ischemic stroke. In addition, the stroke risk factors of different kinds of TCM were also compared to provide references for the study regarding the cause of recurrence. This article will help researchers to better understand the advantages of TCM in the treatment of stroke and offer better assistance for clinical applications.

## Materials and Methods

Our systematical analysis was registered in the International Platform of Registered Systematic Review and Meta-Analysis Protocols (INPLASY) under the registration number INPLASY202150036. The analysis was carried out according to the Preferred Reporting Items for Systematic Reviews and Meta-Analyses (PRISMA) guidelines (Hutton et al., 2015) (Supplementary Material S1). The abbreviations used in this manuscript have been listed in Supplementary Material S2.

### Eligibility and Exclusion Criteria

The (Patient/Intervention/Comparison/Outcome/Study design) PICOS framework was employed as our eligibility criteria, thus only randomized controlled trials (RCTs) consistent with the following requirements were taken into our consideration: (1) Participants: patients with the diagnosis of ischemic stroke with no limitation on nationality, race, gender, age, and disease duration; (2) Interventions and comparisons: the treatment group was given conventional WM plus TCM, and the control group adopted another kind of TCM plus WM or WM alone. In addition, the WM treatment must be the same between treatment and control groups. The common WM drugs were antiplatelet, statins, antihypertensive drugs, hypoglycemic drugs, etc. whereas TCM included NXT, TXL, BUHW, NMT, DZSM, NST, and MXK. Besides, there were no limitations on dosage and the follow-up time was defined as more than 2 months; (3) Outcomes: the primary outcomes of this systematical analysis were recurrent stroke and the National Institute of Health stroke scale (NIHSS). Additionally, the secondary outcomes were stroke risk factors including elevated fibrinogen (Fib), fasting blood glucose (FBG), triglycerides (TG), and total cholesterol (TC). Moreover, the safety outcomes were all-cause mortality and adverse events (AEs). In this systematical analysis, RCT which included one of the primary outcomes was sufficient. Furthermore, the reasons behind choosing these secondary outcomes are the fact that increases in Fib (Refaai et al., 2018), FBG (Lawes et al., 2004), TG (Yuan et al., 2020), and TC (Yuan et al., 2020) have a close association with ischemic stroke or cardiovascular events; and (4) Study design: Only RCTs were taken into consideration for the current investigation.

RCTs would be excluded if they were in accordance with the following criteria: (1) The follow-up time was less than 2 months; (2) the treatment methods include acupuncture or other kinds of TCM; (3) publications were duplicated; (4) data was incomplete; (5) there were no relevant outcomes; and (6) the patients had serious complications.

### Search Strategy

PubMed, Web of Science, embase, China National Knowledge Infrastructure (CNKI), Chinese Biological Medicine Literature Service System (CBM), China Science and Technology Journal database (VIP), and Wan−fang database (WF) were searched for this investigation. The included studies were published in the period starting from the foundation of each database to May 5, 2021. The topic search used was the combination of medical topic title terminology and free text terminology. Searched terms included “ischemic stroke,” “cerebral infarction,” “secondary prevention,” “Naoxintong Capsule,” “Tongxinluo Capsule,” “Buyang Huanwu Decoction,” “Naomaitai Capsule,” “Dengzhan Shengmai Capsule,” “Naoshuantong Capsule,” and “Maixuekang Capsule”. The detailed search process of PubMed is outlined in Table 1. Furthermore, references in the literature were searched manually.

**TABLE 1:** The detailed search process of PubMed.

Serial number	Strategy
#1	Ischemic stroke (MeSH Terms) OR cerebral infarction (MeSH Terms)
#2	Cerebral Infarctions (Title/Abstract)) OR Infarctions, Cerebral (Title/Abstract)) OR Infarction, Cerebral (Title/Abstract)) OR Cerebral Infarction, Left Hemisphere (Title/Abstract)) OR Left Hemisphere
	Infarction, Cerebral (Title/Abstract)) OR Infarction, Left Hemisphere, Cerebral (Title/Abstract)) OR Left
	Hemisphere, Cerebral Infarction (Title/Abstract)) OR Cerebral, Left Hemisphere, Infarction (Title/Abstract)) OR Infarction, Cerebral, Left Hemisphere(Title/Abstract)) OR Subcortical Infarction(Title/Abstract)
#3	#1 AND #2
#4	secondary prevention (Title/Abstract)
#5	Naoxintong Capsule (Title/Abstract)
#6	Tongxinluo Capsule (Title/Abstract)
#7	Buyang Huanwu Decoction (Title/Abstract)
#8	Naomaitai Capsule (Title/Abstract)
#9	Dengzhan Shengmai Capsule (Title/Abstract)
#10	Naoshuantong Capsule (Title/Abstract)
#11	Maixuekang Capsule (Title/Abstract)
#12	AND/#5−#11
#13	randomized controlled trial (Publication Type) OR controlled clinical trial (Publication Type)
#14	randomized (Title/Abstract) OR placebo (Title/Abstract) OR randomly (Title/Abstract)
#15	#13 OR #14
#16	animals (MeSH Terms)
#17	humans (MeSH Terms)
#18	#16 NOT #17
#19	#12 NOT #18
#20	#3 AND #4 AND #12 AND #19

### Literature Inclusion and Data Extraction

Endnote 20.0 software was utilized for creating a library of the obtained articles and the literature inclusion was done by two researchers independently. Duplicated articles were excluded first. The titles and abstracts of articles were then thoroughly read for primary screening in accordance with the inclusion and exclusion criteria. The next step involved the re-screening of the articles via reading full-text content based on the inclusion and exclusion criteria. A third researcher would additionally participate in the discussion if at any stage the results were controversial. Moreover, relevant data were extracted which included publication date, author’s name, title, detailed characteristics of participants (sample capacity, age, sex), interventions (drug, dose, and follow-up time), outcomes (primary outcomes, secondary outcomes, and safety outcomes), and elements which were used to evaluate the risk of bias. Furthermore, since our research was related to ethnopharmacology, the compositions of included medicines were clearly recorded (Heinrich et al., 2020). In addition, all botanical plants were named according to the existing international standards in databases (Rivera et al., 2014).

### Risk of Bias Assessment

According to the Cochrane risk of bias tool (Higgins et al., 2011), the quality assessment of all included RCTs was conducted by two researchers independently. Every RCT was classified as low, unclear, or high risk of bias based on seven quality evaluation items precisely: selection bias (random sequence generation and allocation concealment), performance bias (blinding of participants and personnel), detection bias (blinding of outcome assessment), attrition bias (incomplete outcome data), reporting bias (selective reporting), and other bias. In addition, when assessing outcomes, the objective outcomes (e.g., recurrent stroke) and subjective outcomes (e.g., NIHSS) were considered separately. When a discordance exists between two researchers, the ultimate outcomes would be resolved by consensus with a third researcher.

### Statistical Analysis

For binary variables, the outcomes were shown as odds ratios (ORs) as well as 95% confidence intervals (95% CIs). For continuous variables, the outcomes were presented as the mean differences (MDs) as well as 95% CIs. Moreover, the results were significantly different when 95% CIs of ORs did not contain 1 or 95% CIs of MDs did not include 0. In addition, if each outcome had at least two studies, the pairwise meta-analysis would be performed with a random effects model. Besides, different interventions would be compared by network meta-analysis under a frequentist framework with a random effects model. League tables were also utilized to display the findings of the systematical analysis. The chance of each treatment included in the systematical analysis being the best was evaluated using the surface under the cumulative ranking curve area (SUCRA) to obtain the best treatment (Salanti et al., 2011). In addition, cluster analysis was used to determine the optimum therapy for ischemic stroke.

In the standard pairwise meta-analysis, the statistical heterogeneity was tested by the calculation of I^2^ statistics and the clinical heterogeneity was assessed by comparisons of data on potential effect modifiers. If the I^2^ ≤ is 50%, the heterogeneity is not obvious (Higgins et al., 2003). Since each network graph was not looped, the incoherence (the statistical disagreement between direct and indirect results) could not be evaluated. In systematical analysis, we also assumed a consistent estimate for the heterogeneity variance. The distribution of putative effect modifiers was examined to determine transitivity between treatment comparisons (Turner et al., 2012).

If the number of studies was adequate, funnel plots were used to determine the presence of publication bias. Furthermore, the GRADE evaluation would serve as the foundation for the quality assessment, which would cover the five aspects of research restriction: study limitation, indirectness, inconsistency, imprecision, and publication bias (Salanti et al., 2014). A subgroup meta-analysis was also performed to evaluate the following possible effect modifiers (source of heterogeneity): 1. Treatment dosage (low dose, median dose, and high dose). 2. The age of patients (mean age less than 60 and mean age not less than 60). 3. The specific methods of WM (with statins and without statins). Finally, a post hoc sensitivity analysis was made: we used a leave−one−out meta-analysis to identify the independent impact of each study on the pooled estimates. We use Stata 16.0 software to get the statistical outcomes and statistical graphing of this systematical analysis.

## Results

### Literature Selection

**FIGURE 1:**
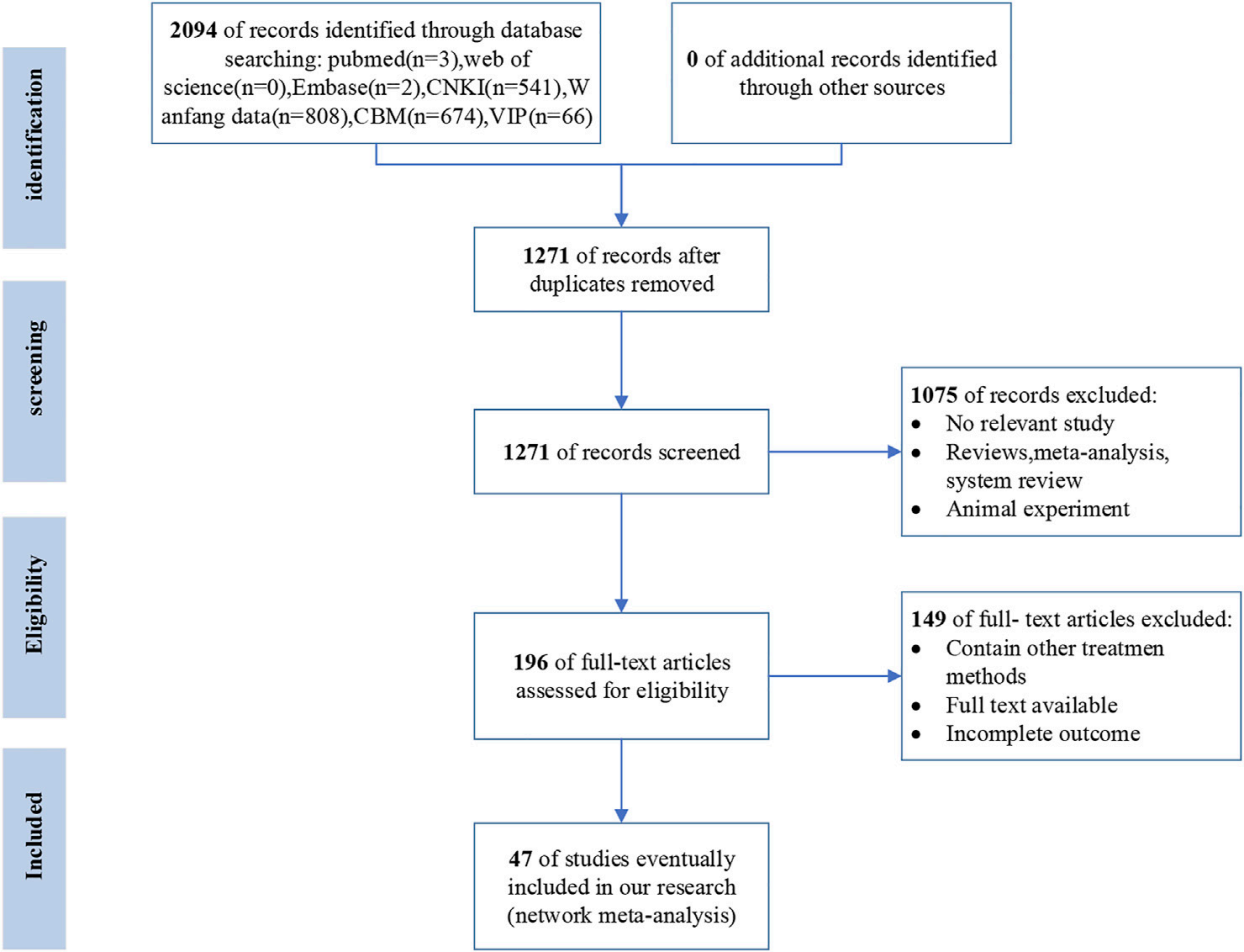
Flow diagram of literature screening (CNKI, National Knowledge Infrastructure; CBM, the Chinese Biological Medicine Literature Service System; VIP, the Chinese Scientific Journal Full−text database; WF, the Wan−fang database; n, number of articles).

A total of 2094 articles were obtained at first, including 541 articles from CNKI, 808 articles from WanFang data, 674 articles from CBM, 66 articles from VIP, 3 articles from PubMed, 0 and 2 articles, respectively, from the web of science and embase. After de-duplicating articles, a total of 1271 articles were taken into consideration. Then, following a thorough reading of the title and abstract of each publication, we excluded the publications that were not relevant to our systematical analysis. Furthermore, the full text of the remaining 196 articles was carefully read to find articles that met our PICOS principles. In the end, 47 articles were taken into consideration. In conclusion, only RCTs about the comparison between TCM + WM and WM were ultimately chosen. Furthermore, the conditions of patients and treatment methods must meet our pre-determined requisites. Moreover, the outcomes in the literature must have one of the primary outcomes. In addition, the follow-up time must be longer than 2 months. We show the PRISMA diagram about the further details of the article screening process in Figure 1.

### Study Characteristics

**FIGURE 2:**
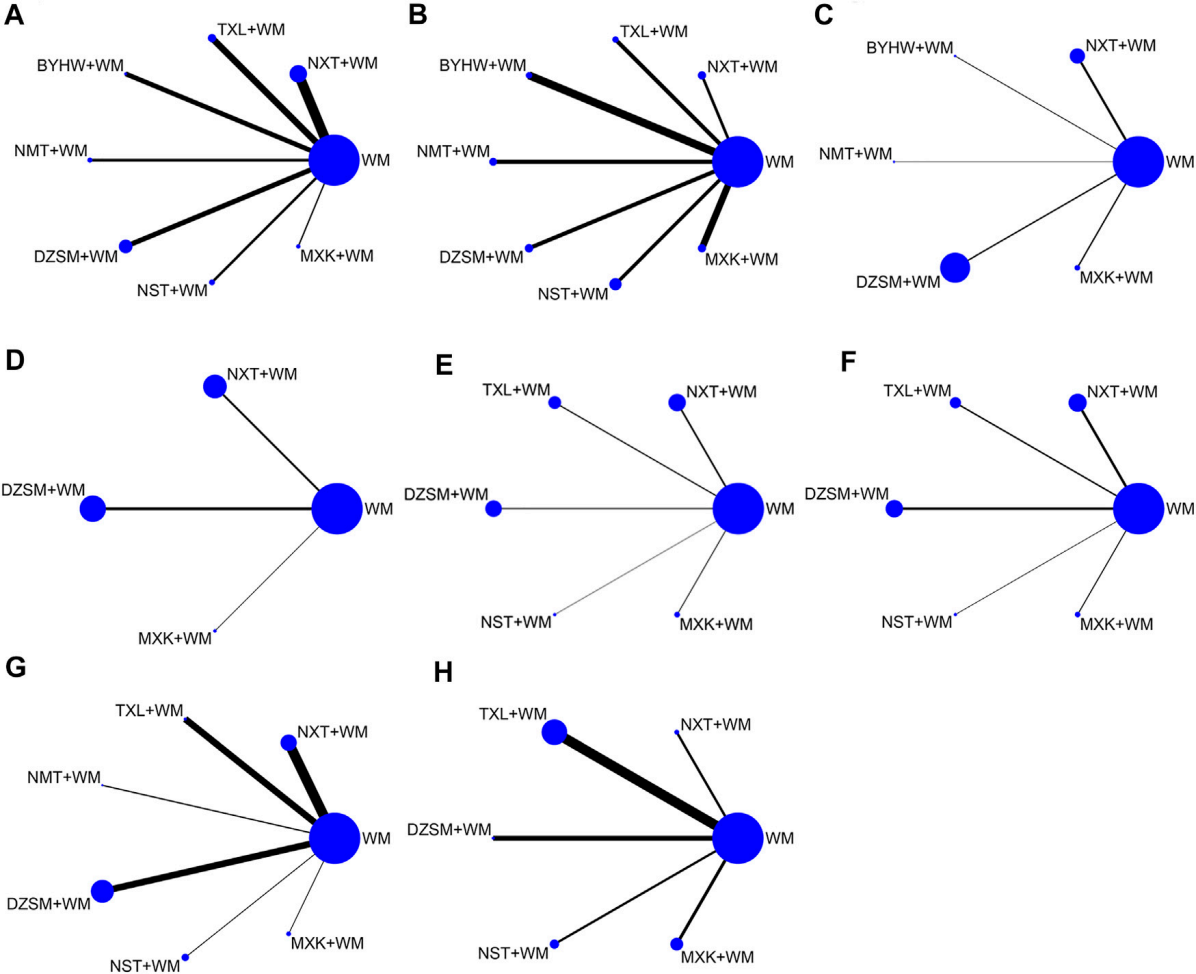
Network graphs for various outcomes. The size of the point is assigned by the total number of people in each study, and the width of the edge is assigned by standard error. **(A)** Recurrent stroke; **(B)** NIHSS; **(C)** Fib; **(D)** FBG; **(E)** TG; **(F)** TC; **(G)** all−cause mortality; **(H)** AEs (NXT, Naoxintong capsule; TXL, Tongxinluo capsule; BYHW, Buyang Huanwu Decoction; NMT, Naomaitai capsule; DZSM, Dengzhan Shengmai capsule; NST, Naoshuantong capsule; MXK, Maixuekang capsule; WM, conventional Western medicine; Fib, fibrinogen; FBG, fasting plasma glucose; TG, triglycerides; TC, cholesterol; AEs, adverse events).

On the whole, there were 47 RCTs (Liu and Wang, 2006; Gan, 2007; Huang and Huang, 2007; Chen et al., 2008; Liu and Cao, 2008; Sun, 2008; Yan et al., 2008; Zhang et al., 2008; Liu, 2009; Meng, 2009; Tian and Li, 2010; Shi D. H. et al., 2011; Shi F. H. et al., 2011; Chao, 2011; Jiao et al., 2011; Zang et al., 2011; Chen, 2012; Jiang, 2012; Lin and Li, 2012; Peng et al., 2012; Guo et al., 2013; Tu et al., 2013; Yu, 2013; Zhou, 2013; Huang et al., 2014; Liu et al., 2014; Ma et al., 2014; Peng and Niu, 2014; Song, 2014; Xue et al., 2014; Zhou, 2014; Chai, 2015; Ye et al., 2015; Zhen, 2015; Chen and Cao, 2016; Ge et al., 2016; Nan and Li, 2016; Bo et al., 2017; Xu, 2017; Dang et al., 2018; Lu et al., 2018; Wang, 2018; Wu and Xiang, 2018; Zhou, 2018; Liu, 2019; Sun, 2020) involving 10,732 patients. Most of the patients involved in the trial were middle-aged and elder. All articles were about two-arm comparisons between TCM + WM and WM, and seven comparisons were made a part of our systematical analysis: NXT + WM vs. WM (n = 15), TXL + WM vs. WM (n = 9), BYHW + WM vs. WM (n = 5), NMT + WM vs. WM (n = 5), DZSM + WM vs. WM (n = 5), NST + WM vs. WM (n = 4), MXK + WM vs. WM (n = 4). Moreover, the most used drugs were aspirin, statins, and antihypertensive drugs. The median follow-up time was found to be 1 year (range, 2 months to 5 years). More details of the included articles are described in Table 2. Additionally, the specific intervention methods of TCM in each publication have been demonstrated in Supplementary material S3 Other detail information can be found in Supplementary material S4 which demonstrated indications and chemical analysis of each TCM and Supplementary material S5 which depicted compositions and extraction procedure of each TCM. Moreover, the network graphs of the above seven kinds of TCM + WM have been listed in Figure 2. About Figure 2, the size of the point is assigned by the total number of people in each study, and the width of the edge is assigned by the standard error.

**TABLE 2:** Characteristic of the articles included in this systematical analysis.

Study ID	Sample size		Age		Sex(M/F)		Interventions		Dose of TCM	Follow−up time	Outcomes
—	T	C	T	C	T	C	T	C			
Sun (2020)	75	75	56.18 ± 1.39	55.92 ± 1.47	43/32	41/34	NXT + WM	WM	3c/t,3t/d	1a	①③⑤⑥⑧
Shi et al. (2011a)	562	512	——	——	——	——	NXT + WM	WM	3c/t,3t/d	5a	①
Shi et al. (2011b)	46	45	62.1 ± 7.9	63.4 ± 8.6	24/22	24/21	NXT + WM	WM	3c/t,3t/d	3 m	②⑤⑥
Zang et al. (2011)	52	46	67 ± 9.3	65 ± 3.6	30/22	27/19	NXT + WM	WM	3c/t,3t/d	2a	①
Tian and Li (2010)	360	357	56.8 ± 7.9	58.2 ± 6.8	222/138	204/153	NXT + WM	WM	3c/t,3t/d	1a	①③④⑤⑥⑧
Jiang (2012)	46	50	58 ± 5	56 ± 6	24/22	28/22	NXT + WM	WM	3c/t,3t/d	2a	①③⑥
Tu et al. (2013)	108	106	59.9 ± 8.4		——	——	NXT + WM	WM	3c/t,3t/d	1a	①④⑧
Dang et al. (2018)	161	158	65.7 ± 10.8	65.9 ± 9.9	114/47	97/61	NXT + WM	WM	3c/t,2t/d	519d	①⑧
Zhang et al. (2008)	80	80	40–78(59)		88/72		NXT + WM	WM	4c/t,3t/d	2a	①
Xu (2017)	40	40	75.3 ± 3.2	76.7 ± 3.1	25/15	24/16	NXT + WM	WM	3c/t,3t/d	3a	①⑦
Zhou (2013)	38	48	41–79(60)		32/34		NXT + WM	WM	4c/t,3t/d	2a	①⑧
Meng (2009)	40	40	41–79(61)		44/36		NXT + WM	WM	4c/t,3t/d	2a	①⑧
Wang (2018)	40	40	55.5 ± 14		——	——	NXT + WM	WM	3c/t,3t/d	1a	①③
Zhou (2018)	70	70	41–83(62.08)	42–83(62.47)	45/25	41/29	NXT + WM	WM	4c/t,3t/d	6 m	②③⑤⑥
Chen (2012)	75	60	60 ± 4.3	58 ± 3.8	42/33	30/34	NXT + WM	WM	3c/t,3t/d	3 m	①⑤⑥
Lu et al. (2018)	35	35	62.4 ± 10.5	61.6 ± 9.4	20/15	18/17	TXL + WM	WM	2c/t,3t/d	6 m	①②⑤⑥⑧
Liu and Cao (2008)	30	30	61 ± 11	63 ± 10	17/13	19/11	TXL + WM	WM	2c/t,3t/d	3a	①⑦⑧
Song (2014)	86	76	58.48 ± 7.79	57.12 ± 7.4	45/41	41/35	TXL + WM	WM	3c/t,3t/d	6 m	①⑦
Bo et al. (2017)	68	77	66.88 ± 10.6	66.99 ± 10.24	32/36	33/44	TXL + WM	WM	4c/t,3t/d	1a	①⑦
Zhou (2014)	200	200	53.1 ± 9.7		268/132		TXL + WM	WM	4c/t,3t/d	1a	①⑤⑥
Yan et al. (2008)	182	178	——		——		TXL + WM	WM	3c/t,3t/d	1a	①
Chai (2015)	84	84	66.1 ± 7.2	65.8 ± 6.3	53/31	52/32	TXL + WM	WM	3c/t,3t/d	1a	⑤⑥
Xue et al. (2014)	74	74	66.1 ± 7.2	65.8 ± 6.3	42/32	43/31	TXL + WM	WM	4c/t,3t/d	1a	①⑤⑥⑦
Guo et al. (2013)	50	48	59.7 ± 5.4	60.4 ± 5.1	39/11	36/12	TXL + WM	WM	2c/t,3t/d	1a	①②⑤⑧
Jiao et al. (2011)	49	53	——		——		BYHW + WM	WM	1p/d	6 m	①②
Yu (2013)	49	53	——		——		BYHW + WM	WM	1p/d	6 m	①
Lin and Li (2012)	37	37	47–83	49–81	20/17	19/18	BYHW + WM	WM	1p/d	2a	①
Liu (2019)	24	24	56 ± 6.5	54.8 ± 6.8	15/9	14/10	BYHW + WM	WM	1p/d	3 m	①③
Chen and Cao (2016)	35	35	42–75		39/31		BYHW + WM	WM	1p/d	6 m	①②③
Sun (2008)	50	50	72.7 ± 10.2	71.3 ± 11.6	26/24	25/25	NMT + WM	WM	2c/t,3t/d	2a	①
Huang and Huang (2007)	218	200	62.46 ± 6.28	60.35 ± 6.73	143/75	131/69	NMT + WM	WM	2c/t,3t/d	3a	①
Liu and Wang (2006)	80	80	65.28 ± 13.12	64.41 ± 12.56	60/40	54/46	NMT + WM	WM	2c/t,3t/d	3a	①
Gan (2007)	58	60	48–75	45–78	33/25	38/22	NMT + WM	WM	2c/t,3t/d	3 m	①②⑧
Liu et al. (2014)	50	50	61.6 ± 8.6	63.3 ± 7.7	31/19	29/21	NMT + WM	WM	2c/t,3t/d	2 m	②③
Liu (2009)	21	21	57.9 ± 9.14	55.41 ± 9.42	10/11	8/13	DZSM + WM	WM	2c/t,2t/d	6 m	①③④⑤⑥⑦⑧
Nan and Li (2016)	63	63	61.94 ± 10.96	62.42 ± 10.17	35/28	36/27	DZSM + WM	WM	2c/t,3t/d	6 m	①②③⑤⑥⑧
Chao (2011)	620	620	60.82 ± 9.04	61.44 ± 8.77	225/395	215/405	DZSM + WM	WM	2c/t,2t/d	1a	①③⑧
Chen et al. (2008)	495	504	63.3 ± 9.1	66.3 ± 8.0	241/254	267/237	DZSM + WM	WM	2c/t,3t/d	1.5a	①③④⑤⑥⑧
Ma et al. (2014)	55	55	60.6 ± 5.8	60.9 ± 5.4	30/25	31/24	DZSM + WM	WM	2c/t,3t/d	6 m	①②⑥
Liu and Li (2012)	87	86	69 ± 5.04	68.2 ± 5.05	52/35	53/33	NST + WM	WM	3c/t,3t/d	1a	①②⑤⑥⑦
Ye et al. (2015)	345	352	62.37 ± 9.97	62.82 ± 9.97	232/113	231/121	NST + WM	WM	3c/t,3t/d	6 m	①⑧
Huang et al. (2014)	49	45	68.5 ± 6.3	68.7 ± 6.5	29/20	27/18	NST + WM	WM	3c/t,3t/d	3 m	①②
Peng et al. (2012)	45	45	60.26 ± 9.52	61.75 ± 10.26	22/23	26/19	NST + WM	WM	3c/t,3t/d	6 m	②
Ge et al. (2016)	200	198	——	——	——	——	MXK + WM	WM	3c/t,3t/d	1a	①⑧
Peng and Niu(2014)	124	124	65 ± 14	64 ± 13	69/55	67/57	MXK + WM	WM	2c/t,3t/d	1a	①③⑤⑥⑦
Zhen (2015)	49	49	70.22 ± 7.33	70.12 ± 7.19	27/22	28/21	MXK + WM	WM	3c/t,3t/d	3 m	②④⑤⑥
Wu and Xiang (2018)	65	65	62.3 ± 4.3	61.2 ± 4.1	34/31	36/29	MXK + WM	WM	3c/t,3t/d	3 m	②③

T, treatment group; C, control group; M, male; F, Female; NXT, Naoxintong capsule; TXL, Tongxinluo capsule; BYHW, Buyang Huanwu Decoction; NMT, Naomaitai capsule; DZSM, Dengzhan Shengmai capsule; NST, Naoshuantong capsule; MXK, Maixuekang capsule; WM, conventional Western medicine; c, capsule; p, package; t, time; d, day; m, month; a, year; ①, recurrent stroke; ②, NIHSS; ③, fibrinogen; ④, fasting blood glucose; ⑤, triglycerides; ⑥, total cholesterol; ⑦, all−cause mortality; ⑧, adverse events.

### Risk of Bias Assessment

**FIGURE 3:**
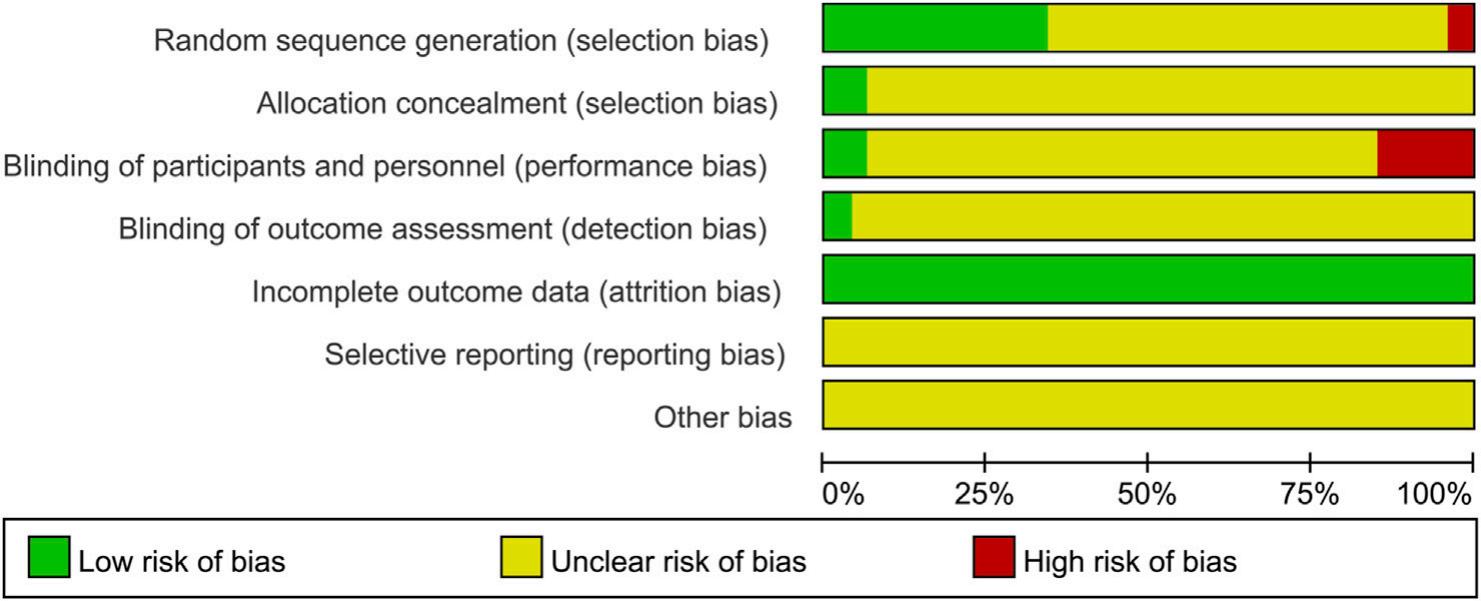
Summary of the risk of bias. The vertical axis represents the quality evaluation items, and the horizontal axis represents the number of randomized controlled trials. Herein green indicates low risk of bias, yellow indicates medium risk of bias, and red indicates high risk of bias.

When it comes to random sequence generation, 16 articles used random number tables or other random methods, therefore these articles were presumed to have a low risk of bias. On the contrary, two articles generated random sequences according to the admission time, which will lead to a high risk of bias. Regarding allocation concealment, three RCTs highlighted that random sequences would be kept by a third party; they were thus evaluated to have a low risk of bias. In terms of performance bias, two articles mentioned the performance of the double-blind method leading to a low risk of bias. Nevertheless, seven articles did not use a placebo, thus the evaluation was “high.” In terms of blinding of outcome assessment, two articles were assessed to have a low risk for using the double-blind method. Moreover, since all articles had no incomplete data, we classify them as low risk. Due to the lack of sufficient information, the biased entries of other aspects in each article were evaluated to be “unclear.” In conclusion, the quality of included articles was poor. Summary of the risk of bias is demonstrated in Figure 3 in which green indicates a low risk of bias, yellow indicates a medium risk of bias, and red indicates a high risk of bias.

### Outcomes

#### Recurrent Stroke

In total, 40 studies involving 7 treatments regarding recurrent stroke were evaluated: NXT + WM vs. WM (n = 13), TXL + WM vs. WM (n = 8), BYHW + WM vs. WM (n = 5), NMT + WM vs. WM (n = 4), DZSM + WM vs. WM (n = 5), NST + WM vs. WM (n = 3), MXK + WM vs. WM (n = 2). All types of TCM + WM were superior to WM alone, and the results were significantly different. Table 3 shows the detailed information of comparisons: NXT + WM vs. WM (OR = 0.54, 95%CIs (0.43, 0.66)), TXL + WM vs. WM (OR = 0.44, 95%CIs (0.31, 0.61)), BYHW + WM vs. WM (OR = 0.48, 95%CIs (0.26, 0.89)), NMT + WM vs. WM (OR = 0.20, 95%CIs (0.13, 0.32)), DZSM + WM vs. WM (OR = 0.53, 95%CIs (0.36, 0.79)), NST + WM vs. WM (OR = 0.36, 95%CIs (0.19, 0.68)), MXK + WM vs. WM (OR = 0.33, 95%CIs (0.16, 0.68)). Furthermore, NMT + WM vs. NXT + WM (OR = 0.37, 95%CIs (0.22, 0.62)), NMT + WM vs. TXL + WM (OR = 0.46, 95%CIs (0.26, 0.82)), NMT + WM vs. BYHW + WM (OR = 0.42, 95%CIs (0.19, 0.91)), NMT + WM vs. DZSM + WM (OR = 0.38, 95%CIs (0.20, 0.69)) were related to significantly reduce recurrent stroke. Other comparisons did not have statistically significant differences.

**TABLE 3:** Final results of the systematical meta−analysis.

Outcomes	WM	NXT + WM	TXL + WM	BYHW + WM	NMT + WM	DZSM + WM	NST + WM	MXK + WM
Recurrent stroke (OR)	WM	——	——	——	——	——	——	——
	1.86 (1.50,2.30)	NXT + WM	——	——	——	——	——	——
	2.29 (1.63,3.22)	1.23 (0.82,1.84)	TXL + WM	——	——	——	——	——
	2.09 (1.12,3.90)	1.12 (0.58,2.17)	0.91 (0.45,1.86)	BYHW + WM	——	——	——	——
	5.00 (3.13,7.97)	2.68 (1.61,4.49)	2.18 (1.22,3.89)	2.39 (1.10,5.22)	NMT + WM	——	——	——
	1.87 (1.26,2.79)	1.01 (0.64,1.58)	0.82 (0.48,1.38)	0.90 (0.43,1.88)	0.38 (0.20,0.69)	DZSM + WM	——	——
	2.79 (1.47,5.29)	1.50 (0.76,2.94)	1.22 (0.59,2.51)	1.34 (0.55,3.27)	0.56 (0.25,1.23)	1.49 (0.70,3.16)	NST + WM	——
	2.99 (1.47,6.07)	1.61 (0.77,3.36)	1.30 (0.59,2.86)	1.43 (0.56,3.68)	0.60 (0.26,1.40)	1.59 (0.71,3.59)	1.07 (0.41,2.78)	MXK + WM
NIHSS (WM)	WM	——	——	——	——	——	——	——
	2.91 (2.43,3.39)	NXT + WM	——	——	——	——	——	——
	1.40 (0.61,2.19)	−1.51 (−2.43,−0.58)	TXL + WM	——	——	——	——	——
	3.51 (1.91,5.11)	0.60 (−1.07,2.27)	2.11 (0.33,3.89)	BYHW + WM	——	——	——	——
	1.37 (0.60,2.15)	−1.53 (−2.44,−0.62)	−0.03 (−1.13,1.08)	−2.14 (−3.92,−0.36)	NMT + WM	——	——	——
	1.62 (0.85,2.38)	−1.29 (−2.19,−0.39)	0.21 (−0.88,1.31)	−1.90 (−3.67,−0.12)	0.24 (−0.85,1.33)	DZSM + WM	——	——
	2.47 (2.31,2.63)	−0.44 (−0.94,0.07)	1.07 (0.27,1.87)	−1.04 (−2.65,0.57)	1.10 (0.30,1.89)	0.86 (0.08,1.63)	NST + WM	——
	2.63 (1.24,4.03)	−0.27 (−1.74,1.20)	1.23 (−0.37,2.83)	−0.88 (−3.00,1.24)	1.26 (−0.33,2.85)	1.02 (−0.57,2.61)	0.16 (−1.24,1.56)	MXK + WM
Fib (WM)	WM	——	——	——	——	——	——	——
	1.04 (0.86,1.22)	NXT + WM	——	——	——	——	——	——
	1.09 (0.83,1.35)	0.05 (−0.26,0.36)	——	BYHW + WM	——	——	——	——
	0.09 (−0.31,0.49)	−0.95 (−1.39,−0.51)	——	−1.00 (−1.48,−0.52)	NMT + WM	——	——	——
	0.28 (0.10,0.47)	−0.76 (−1.02,−0.50)	——	−0.81 (−1.13,−0.49)	0.19 (−0.25,0.63)	DZSM + WM	——	——
	1.28 (0.93,1.62)	0.24 (−0.16,0.63)	——	0.19 (−0.25,0.62)	1.19 (0.66,1.72)	1.00 (0.61,1.39)	——	MXK + WM
FBG (WM)	WM	——	——	——	——	——	——	——
	0.03 (−0.13,0.18)	NXT + WM	——	——	——	——	——	——
	0.19 (−0.01,0.40)	0.17 (−0.09,0.42)	——	——	——	DZSM + WM	——	——
	0.41 (0.09,0.73)	0.38 (0.03,0.74)	——	——	——	0.22 (−0.17,0.60)	——	MXK + WM
TG (WM)	WM	——	——	——	——	——	——	——
	0.38 (0.18,0.58)	NXT + WM		——	——	——	——	——
	0.22 (0.03,0.41)	−0.16 (−0.43,0.12)	TXL + WM	——	——	——	——	——
	0.03 (−0.23,0.28)	−0.35 (−0.68,−0.02)	−0.19 (−0.51,0.12)	——	——	DZSM + WM	——	——
	−0.42 (−0.89,0.05)	−0.80 (−1.31,−0.29)	−0.64 (−1.15,−0.14)	——	——	−0.45 (−0.98,0.09)	NST + WM	——
	0.11 (−0.22,0.43)	−0.27 (−0.65,0.11)	−0.12 (−0.49,0.26)	——	——	0.08 (−0.33,0.49)	0.53 (−0.04,1.10)	MXK + WM
TC (WM)	WM	——	——	——	——	——	——	——
	0.85 (0.37,1.34)	NXT + WM	——	——	——	——	——	——
	0.67 (0.08,1.26)	−0.18 (−0.95,0.58)	TXL + WM	——	——	——	——	——
	0.39 (−0.22,1.00)	−0.47 (−1.24,0.31)	−0.28 (−1.13,0.57)	——	——	DZSM + WM	——	——
	−1.49 (−2.68,−0.29)	−2.34 (−3.63,−1.05)	−2.16 (−3.49,−0.82)	——	——	−1.87 (−3.21,−0.53)	NST + WM	——
	0.54 (−0.30,1.39)	−0.31 (−1.29,0.66)	−0.13 (−1.16,0.90)	——	——	0.15 (−0.89,1.20)	2.03 (0.56,3.49)	MXK + WM
All-Cause mortality (OR)	WM	——	——	——	——	——	——	——
	2.63 (1.27,5.45)	NXT + WM	——	——	——	——	——	——
	2.44 (0.43,13.88)	0.93 (0.14,6.11)	TXL + WM	——	——	——	——	——
	0.63 (0.10,4.07)	0.24 (0.03,1.77)	0.26 (0.02,3.31)	——	NMT + WM	——	——	——
	3.07 (1.42,6.60)	1.17 (0.40,3.37)	1.26 (0.19,8.40)	——	4.85 (0.65,36.29)	DZSM + WM	——	——
	1.58 (0.48,5.14)	0.60 (0.15,2.40)	0.65 (0.08,5.29)	——	2.49 (0.28,22.61)	0.51 (0.13,2.10)	NST + WM	——
	1.70 (0.57,5.06)	0.65 (0.17,2.40)	0.70 (0.09,5.43)	——	2.69 (0.31,23.27)	0.55 (0.15,2.10)	1.08 (0.22,5.39)	MXK + WM
AEs (OR)	WM	——	——	——	——	——	——	——
	2.62 (0.36,19.24)	NXT + WM	——	——	——	——	——	——
	1.51 (0.57,4.01)	0.58 (0.06,5.32)	TXL + WM	——	——	——	——	——
	1.00 (0.04,23.62)	0.38 (0.01,16.07)	0.66 (0.02,18.15)	——	——	DZSM + WM	——	——
	3.27 (0.47,22.60)	1.25 (0.08,20.11)	2.17 (0.25,18.92)	——	——	3.27 (0.08,133.13)	NST + WM	——
	0.66 (0.07,6.46)	0.25 (0.01,5.23)	0.44 (0.04,5.24)	——	——	0.66 (0.01,32.60)	0.20 (0.01,4.01)	MXK + WM

The bolded and underlined results express statistically significant difference (NXT, Naoxintong capsule; TXL, Tongxinluo capsule; BYHW, Buyang Huanwu Decoction; NMT, Naomaitai capsule; DZSM, Dengzhan Shengmai capsule; NST, Naoshuantong capsule; MXK, Maixuekang capsule; WM, conventional Western medicine).

**FIGURE 4:**
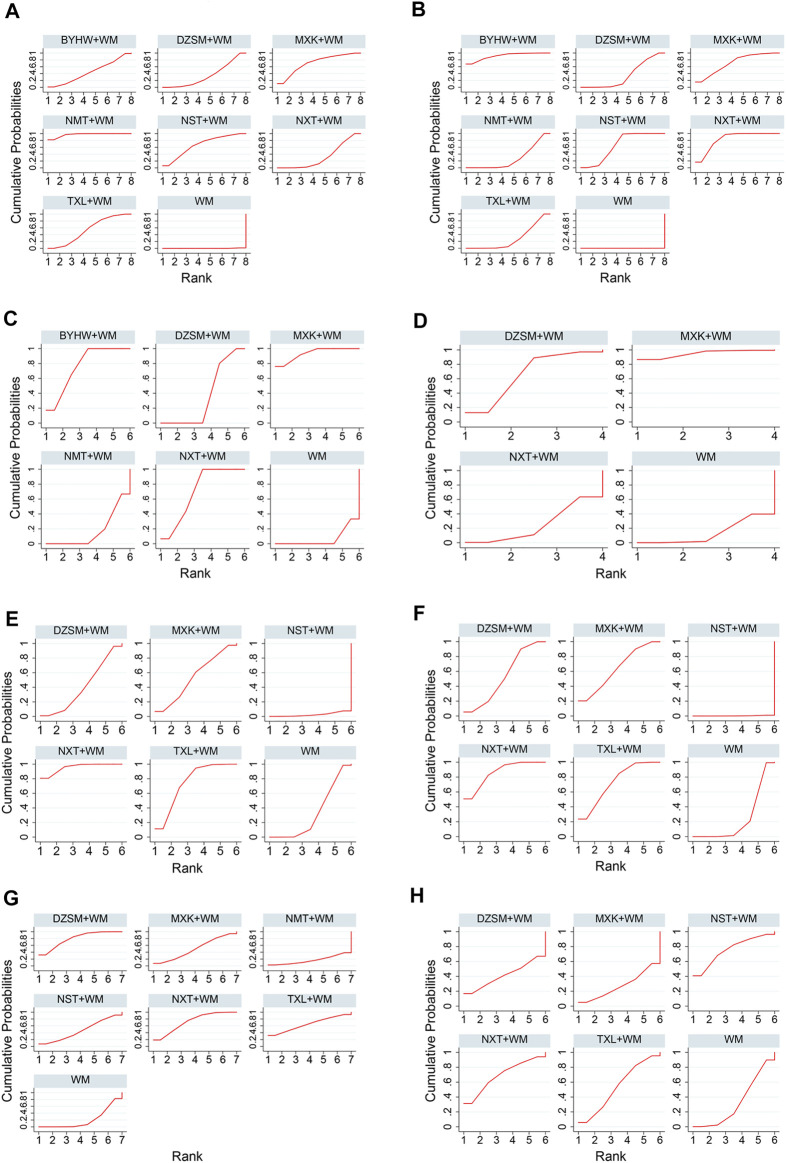
SUCRA for various outcomes. The vertical axis represents cumulative probabilities, and the horizontal axis represents ranking of the treatment. **(A)** Recurrent stroke; **(B)** NIHSS; **(C)** Fib; **(D)** FBG; **(E)** TG; **(F)** TC; **(G)** All-cause mortality; **(H)** AEs (NXT, Naoxintong capsule; TXL, Tongxinluo capsule; BYHW, Buyang Huanwu Decoction; NMT, Naomaitai capsule; DZSM, Dengzhan Shengmai capsule; NST, Naoshuantong capsule; MXK, Maixuekang capsule; WM, conventional Western medicine; Fib, fibrinogen; FBG, fasting blood glucose; TG, triglycerides; TC, cholesterol; AEs, adverse events).

Based on the outcomes of SUCRA probabilities (Table 4; Figure 4), NMT + WM was most likely to be the best choice. In addition, the detailed ranking results of those seven kinds of TCM + WM were depicted as follows: NMT + WM (97.0%) > MXK + WM (71.1%) > NST + WM (66.5%) > TXL + WM (54.1%) > BYHW + WM (44.6%) > DZSM + WM (34.5%) > NXT + WM (32.1%) > WM (0.2%).

**TABLE 4:** SUCRA of different treatments for various outcomes.

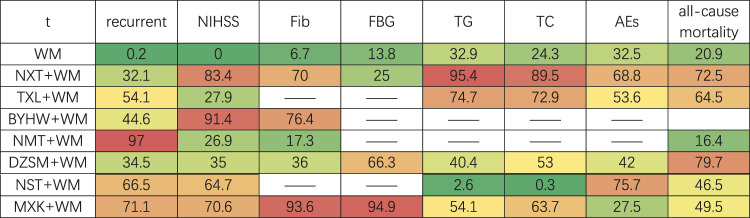

The redder the data, the higher the ranking of the drug in the outcome indicator.

#### NIHSS

A total of 15 researches including 7 interventions referred to NIHSS: NXT + WM vs. WM (n = 2), TXL + WM vs. WM (n = 2), BYHW + WM vs. WM (n = 2), NMT + WM vs. WM (n = 2), DZSM + WM vs. WM (n = 2), NST + WM vs. WM (n = 3), MXK + WM vs. WM (n = 2). Compared with WM alone, all TCM + WM had significant effectiveness regarding NIHSS. In addition, the specific results are listed in Table 3: NXT + WM vs. WM (MD = −2.91, 95%CIs (−3.39, −2.43)), TXL + WM vs. WM (MD = −1.40, 95%CIs (−2.19, −0.61)), BYHW + WM vs. WM (MD = −3.51, 95%CIs (−5.11, −1.91)), NMT + WM vs. WM (MD = −1.37, 95%CIs (−2.15, −0.60)), DZSM + WM vs. WM (MD = −1.62, 95%CIs (−2.38, −0.85)), NST + WM vs. WM (MD = −2.47, 95%CIs (−2.63, −2.31)), MXK + WM vs. WM (MD = −2.63, 95%CIs (−4.03, −1.24)). In addition, comparisons of any one of the three drugs (BYHW + WM, NXT + WM, NST + WM) with any one of the other three drugs (DZSM + WM, NMT + WM, TXL + WM) performed statistically significantly different. Moreover, no statistically significant difference was found between other treatments.

According to the ranking of SUCRA probabilities (Table 4; Figure 4), the order of 7 types of TCM + WM was: BYHW + WM (91.4%) > NXT + WM (83.4%) > MXK + WM (70.6%) > NST + WM (64.7%) > DZSM + WM (35.0%) > TXL + WM (27.9%) > NMT + WM (26.9%) > WM (0.0%).

#### Fib

A total of 14 RCTs involving 5 interventions investigated Fib: NXT + WM vs. WM (n = 5), BYHW + WM vs. WM (n = 2), NMT + WM vs. WM (n = 1), DZSM + WM vs. WM (n = 4), MXK + WM vs. WM (n = 2). Except for NMT + WM, each TCM + WM’s comparison with WM had significant difference. Table 3 specifically expresses the following difference: NXT + WM vs. WM (MD = −1.04, 95%CIs (−1.22, −0.86)), BYHW + WM vs. WM (MD = −1.09, 95%CIs (−1.35, −0.83)), DZSM + WM vs. WM (MD = −0.28, 95%CIs (−0.47, −0.10)), MXK + WM vs. WM (MD = −1.28, 95%CIs (−1.62, −0.93)). Other detailed results can be found in Table 3.

Based on the SUCRA value (Table 4; Figure 4), the ranking of 5 types of TCM + WM was as follows: MXK + WM(93.6%) > BYHW + WM(76.4%) > NXT + WM(70.0%) > DZSM + WM(36%) > NMT + WM(17.3%) > WM(6.7%).

#### FBG

In total, 5 RCTs with three treatments referred to FBG were found: NXT + WM vs. WM (n = 2) DZSM + WM vs. WM (n = 2) MXK + WM vs. WM (n = 1). Only two comparisons in Table 3 showed significant difference: MXK + WM vs. WM (MD = −0.41, 95%CIs (−0.73, −0.09)), MXK + WM vs. NXT + WM (MD = −0.38, 95%CIs: (−0.74, −0.03)). Other results showed no statistically significant difference.

Based on the ranking of SUCRA probabilities (Table 4; Figure 4), the ranking of the included three types of TCM + WM was as follows: MXK + WM (94.9%) > DZSM + WM (66.3%) > NXT + WM (25.0%) > WM (13.8%).

#### TG

A total of 16 articles with 5 interventions presented data about TG: NXT + WM vs. WM (n = 5), TXL + WM vs. WM (n = 5) DZSM + WM vs. WM (n = 3), NST + WM vs. WM (n = 1), MXK + WM vs. WM (n = 2). Compared with WM, only NXT + WM (MD = −0.80, 95%CIs (−1.31, −0.29)) and TXL + WM (MD = −0.64, 95%CIs (−1.15, −0.14)) significantly reduce TG. Also, the comparison results are demonstrated in Table 3.

According to the SUCRA values (Table 4; Figure 4), the order of 5 kinds of TCM + WM was: NXT + WM (95.4%) > TXL + WM (74.7%) > MXK + WM (54.1%) > DZSM + WM (40.4%) > WM (32.9%) > NST + WM (2.6%).

#### TC

The information of TC was available for 17 researches including 5 treatments: NXT + WM vs. WM (n = 6), TXL + WM vs. WM (n = 4) DZSM + WM vs. WM (n = 4), NST + WM vs. WM (n = 1), MXK + WM vs. WM (n = 2). As it is listed in Table 3 that NXT + WM and TXL + WM significantly reduced TC compared with WM alone: NXT + WM vs. WM (MD = −2.34, 95%CIs (−3.63, −1.05)) and TXL + WM vs. WM (MD = −2.16, 95%CIs (−3.49, −0.82)). However, it is interesting to find that compared to WM alone, NST + WM (MD = 1.49, 95%CIs (0.29, 2.68)) significantly improved TC.

According to the ranking of SCURA probabilities (Table 4; Figure 4), the results of ranking of 5 treatments were as follows: NXT + WM (85.9%) > TXL + WM (72.9%) > MXK + WM (63.7%) > DZSM + WM (53%) > WM (24.3%) > NST + WM (0.3%).

#### All-Cause Mortality

In total, 16 studies involving 6 kinds of medicines investigated all-cause mortality: NXT + WM vs. WM (n = 6), TXL + WM vs. WM (n = 3), NMT + WM vs. WM (n = 1), DZSM + WM vs. WM (n = 4), NST + WM vs. WM (n = 1), MXK + WM vs. WM (n = 1). Only two results in Table 3 demonstrate significant difference: DZSM + WM vs. WM (OR = 0.33, 95%CIs (0.15, 0.70)), NXT + WM vs. WM (OR = 0.38, 95%CIs (0.18, 0.79)).

It can be seen from SUCRA values (Table 4; Figure 4) that the six types of TCM + WM’s rankings in reducing all-cause mortality were: DZSM + WM (79.7%) > NXT + WM (72.5%) > TXL + WM (64.5%) > MXK + WM (49.5%) > NST + WM (46.5%) > WM (20.9%) > NMT + WM (16.4%).

#### AEs

Most of the adverse events were gastrointestinal reaction, nausea, and giddiness(Peng and Niu, 2014; Bo et al., 2017; Xu, 2017). Our research showed that patients were tolerable for adverse reactions of TCM + WM or WM. In addition, the information of AEs was available for 8 researches involving 5 treatments: NXT + WM vs. WM (n = 1), TXL + WM vs. WM (n = 4) DZSM + WM vs. WM (n = 1), NST + WM vs. WM (n = 1), MXK + WM vs. WM (n = 1). All results performed no significant differences, which are listed in Table 3 **.**

Based on the ranking of SUCRA probabilities (Table 4; Figure 4), the 5 interventions’ rankings in reducing AEs were: NST + WM (75.7%) > NXT + WM (68.8%) > TXL + WM (53.6%) > DZSM + WM (42.0%) > WM (32.5%) > MXK + WM (27.5%).

### Cluster Analysis

**FIGURE 5:**
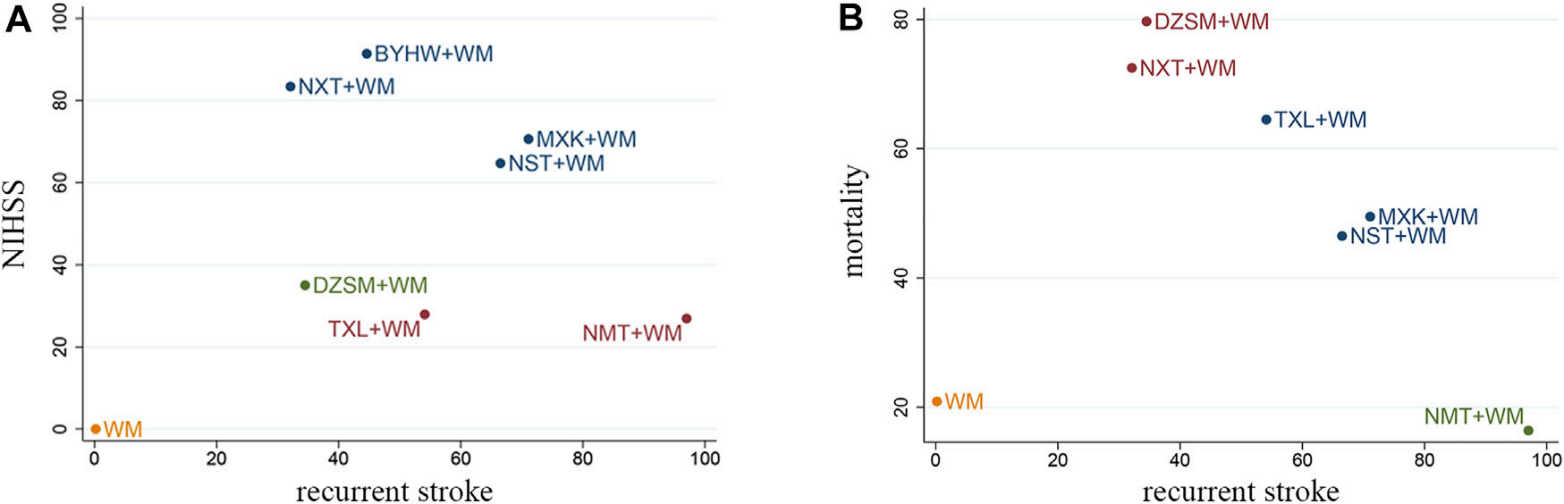
Cluster analysis plots for three outcomes. **(A)** Recurrent stroke (*X* axis), NIHSS (*Y* axis); **(B)** recurrent stroke (*X* axis), all-cause mortality (*Y* axis). Herein blue indicates the best choice considering both outcomes, red indicates the second choice considering both outcomes, green indicates the third choice considering both outcomes, orange indicates the last choice considering both outcomes (NXT, Naoxintong capsule; TXL, Tongxinluo capsule; BYHW, Buyang Huanwu Decoction; NMT, Naomaitai capsule; DZSM, Dengzhan Shengmai capsule; NST, Naoshuantong capsule; MXK, Maixuekang capsule; WM, conventional Western medicine).

Two-dimensional clustering was utilized to find the best TCM through different indicators of various drugs. Herein blue indicates the best choice considering both outcomes, red indicates the second choice considering both outcomes, green indicates the third choice considering both outcomes, and orange indicates the last choice considering both outcomes. When it comes to recurrent stroke and NIHSS, the cluster analysis depicted that BYHW + WM, NXT + WM, MXK + WM, NST + WM were dominant in comparison (Figure 5A). Furthermore, as cluster analysis related to recurrent stroke and al-cause mortality was made, we found that MXK + WM, NST + WM had relatively good curative effects (Figure 5B). In conclusion, the results of cluster analysis demonstrated that MXK + WM, NST + WM had relatively good secondary prevention effects with regard to recurrent stroke, NIHSS, and all-cause mortality.

### Publication Bias

**FIGURE 6:**
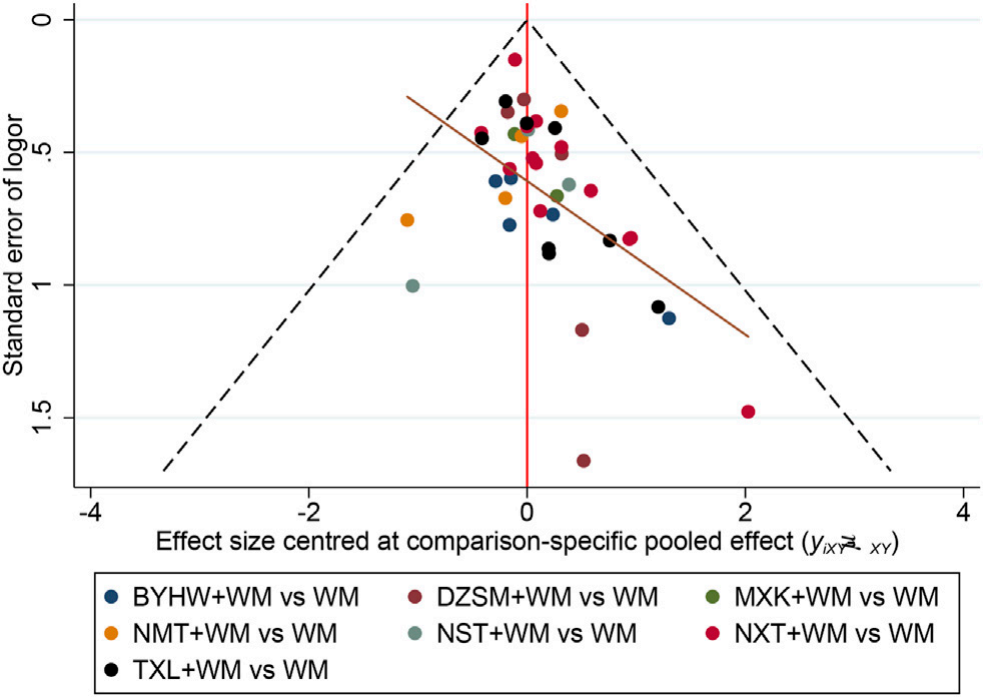
Funnel plot for recurrent stroke. The vertical axis represents “standard error of effect size” and horizontal axis represents “effect size centered at comparison-specific pooled effect.” (NXT, Naoxintong capsule; TXL, Tongxinluo capsule; BYHW, Buyang Huanwu Decoction; NMT, Naomaitai capsule; DZSM, Dengzhan Shengmai capsule; NST, Naoshuantong capsule; MXK, Maixuekang capsule; WM, conventional Western medicine).

To test the publication bias, the comparison−adjusted funnel plots for recurrent stroke were plotted. As it is listed in Figure 6, we found the scatters were basically symmetrical along the center line and the angle between the center line and the adjusted auxiliary line was not large. The results indicated that there is little publication bias and the small sample effects were rare.

### GRADE Assessment

Here, strength of evidence was evaluated through GRADE assessment which was about the comparisons between TCM + WM with WM on recurrent stroke. Except BYHW + WM had high risk of bias leading to low certainty evidence, other types of TCM + WM’s study limitation were uncertain. Furthermore, there was no inconsistency in this systematical analysis which meant no study needs to be downgraded. In addition, all researches had enough sample size and demonstrated obvious tendency to TCM giving rise to no imprecision. Thus, all study didn’t required to be downgraded. Moreover, some of the TCM + WM were downgraded for having publication bias. (Table 5).

**TABLE 5:** GRADE assessment for recurrent stroke.

Summary of GRADE assessment for recurrent stroke					
Comparison effect	Number of direct comparisons	Number of participants	Nature of evidence	Certainty	Reason for down grading
NXT + WM vs. WM	13	1612 vs. 1687	Direct	Low	a, e
TXL + WM vs. WM	8	718 vs. 725	Direct	Moderate	a
BYHW + WM vs. WM	5	202 vs. 194	Direct	Low	b
NMT + WM vs. WM	4	408 vs. 388	Direct	Low	a, e
DZSM + WM vs. WM	5	1263 vs. 1254	Direct	Low	a, e
NST + WM vs. WM	3	483 vs. 480	Direct	Moderate	a
MXK + WM vs. WM	2	327 vs. 327	Direct	Low	a, e

a The quality assessment of most studies is unclear (study limitation).

b The quality assessment of most studies is low (study limitation).

c The heterogeneity between pairwise comparisons was high (inconsistency). [no comparison effect needs to be downgraded for this reason].

d Small sample size or results show no obvious tendency to a drug (imprecision) [no comparison effect needs to be downgraded for this reason].

e Potential publication bias (publication bias).

### Subgroup Analysis

For recurrent stroke and NIHSS, all calculation of potential effect modifier (potential source of heterogeneity) including age of patients (average age <60 vs. average age ≥60), treatment dose (low does vs. medium dose vs. high dose), and the specific methods of WM (with statins vs. without statins) demonstrated no significant difference (Table 6). Thus, we believe that the age of patients and treatment dose had little effect on the transitivity of our systematical analysis.

**TABLE 6:** Subgroup analysis for recurrent stroke and NIHSS.

Outcome	Average age		*p* value for interaction	Dose			*p* value for interaction	Western medicine		*p* value for interaction
	<60	≥60		Low	Median	High		With stain	Without statin	
Placebo(reference)										
Recurrent stroke										
NXT + WM	0.39 (0.18, 0.83)	0.43 (0.29, 0.66)	0.8	0.82 (0.35, 1.88)	0.53 (0.42, 0.69)	0.52 (0.31, 0.89)	0.62	0.52 (0.36, 0.75)	0.55 (0.43, 0.72)	0.79
TXL + WM	0.45 (0.28, 0.73)	0.35 (0.19, 0.64)	0.51	0.29 (0.11, 0.77)	0.46 (0.24, 0.89)	0.36 (0.15, 0.86)	0.74	0.44 (0.31, 0.63)	0.36 (0.06, 2.01)	0.81
BYHW + WM	0.13 (0.01, 1.18)	——	——	——	0.48 (0.26, 0.89)	——	——	0.59 (0.28, 1.22)	0.28 (0.08, 0.92)	0.29
NMT + WM	——	0.16 (0.10, 0.26)	——	0.20 (0.11, 0.36)	——	——	——	0.15 (0.09, 0.26)	0.36 (0.14, 0.97)	0.12
DZSM + WM	——	0.54 (0.36, 0.80)	——	0.54 (0.30, 0.96)	0.60 (0.31, 1.16)	——	0.8	0.60 (0.31, 1.16)	0.50 (0.30, 0.82)	0.64
NST + WM	——	0.32 (0.16, 0.62)	——	——	0.36 (0.19, 0.68)	——	——	0.32 (0.16, 0.62)	1.02 (0.14, 7.31)	0.27
MXK + WM	——	0.33 (0.16, 0.67)	——	0.25 (0.07, 0.94)	0.37 (0.16, 0.86)	——	0.64	0.25 (0.07, 0.94)	——	——
NIHSS										
NXT + WM	−2.91 (−3.44, −2.38)	−2.90 (−3.99, −1.81)	0.99	——	−2.91 (−3.44, −2.38)	−2.90 (−3.99, −1.81)	0.99	−2.91 (−3.39, −2.43)	——	——
TXL + WM	−1.12 (−2.61, 0.37)	−1.51 (−2.44, −0.58)	0.66	−1.40 (−2.19, −0.61)	——	——	——	−1.40 (−2.19, −0.61)	——	——
BYHW + WM	−3.51 (−5.11, −1.91)	——	——	——	−3.51 (−5.11, −1.91)	——	——	−3.51 (−5.11, −1.91)	——	——
NMT + WM	——	−1.37 (−2.15, −0.60)	——	−1.37 (−2.15, −0.60)	——	——	——	−1.22 (−2.10, −0.34)	−1.90 (−3.53, −0.27)	0.47
DZSM + WM	——	−1.89 (−2.18, −1.60)	——	−1.89 (−2.18, −1.60)	——	——	——	−1.90 (−2.78, −1.02)	−0.76 (−2.29, −0.77)	0.2
NST + WM	——	−2.17 (−2.80, −1.53)	——	——	−2.17 (−2.80, −1.53)	——	——	−2.17 (−2.80, −1.53)	——	——
MXK + WM	——	−2.63 (−4.03, −1.24)	——	——	−2.63 (−4.03, −1.24)	——	——	−2.63 (−4.03, −1.24)	——	——

All I^2^<50%.

### Sensitivity Analysis

In the standard pairwise meta-analysis, statistical heterogeneity was tested by the calculation of I^2^ if the number of the study was at least 5 and we find all I^2^<50%. Additionally, a leave−one−out meta-analysis was used to evaluate the independent effect of every study on summary results. The results suggested that no study needed to be excluded, thus we thought each study produced little bias in the results.

## Discussion

Unilateral weakness, unilateral sensory loss, speech disturbance, clumsiness or ataxia, and vertigo are all common stroke symptoms (Hankey and Blacker, 2015). Non-contrast CT is used to precisely diagnose the condition because of its sensitivity and rapidity. RI is also employed to refine the understanding of infarct location (Powers et al., 2019). In addition, most ischemic strokes originated from thromboembolism, the common source of embolism is atherosclerosis and heart disease, especially atrial fibrillation. Other causes of ischemic stroke involve small vessel disease, which has a close association with hypertension and diabetes, especially in Asia (Campbell et al., 2019).

The systematical analysis approach was taken to compare the secondary prevention effects and safety of TCM + WM in the treatment of stroke. In this study, a total of 47 RCTs including 10,732 patients were included in this work, and we evaluated 7 treatments in terms of 8 indicators involving recurrent stroke, NIHSS, Fib, FBG, TG, TC, all-cause mortality, and AEs. The results indicated that all kinds of TCM + WM were more effective on long-term secondary prevention of ischemic stroke than WM alone. Additionally, through cluster analysis, we concluded that MXK + WM and NST + WM had relatively good preventive effects.

In this systematical analysis, seven kinds of TCM were extracted from Astragali Radix, Radix, Chuanxiong Rhizoma, Persicae Semen, Carthami Flos, Pheretima, or other herbs through modern technological means (Bu et al., 2020). Guo et al. reported TCM has the effects of anti-oxidation, anti-inflammatory, anti-apoptosis, and protection of blood−brain barrier (Guo et al., 2017). Furthermore, Yan et al. found that the TCM can prevent stroke through a variety of signaling pathways (Yan et al., 2019), such as JAK2/STAT3 which regulates proinflammatory cytokine expression (Chen et al., 2017; Hu et al., 2017), NF−κB which is related to the inflammatory mechanism of brain tissue reperfusion injury (Fann et al., 2018; Fu et al., 2020), PI3K/Akt which participates in the pathological process of cerebral ischemia and plays a role in promoting survival and anti-apoptotic pathway (Samakova et al., 2019; Chen et al., 2020).

Nevertheless, there were few systematic reviews and meta-analyses to show the long-term secondary preventive effects of TCM on ischemic stroke. The comparisons between them were rather ambiguous leading to confusion in choosing the appropriate drugs. Thus, this study used pairwise meta-analysis as well as network meta-analysis thus better demonstrating the effects of different kinds of TCM. In addition, different types of TCM were quantitatively ranked to determine the best choice for treatment in clinical settings.

In terms of primary outcomes including recurrent stroke and NIHSS, the results suggested that all types of TCM + WM were better than WM, and results had a statistically significant difference. Some articles reported its origin from multi-channel and multi-target therapy of TCM (Li and Li, 2016; Wei, 2016). Wang et al. said NXT can decrease recurrent stroke and NIHSS, which also corroborates well with our study (Wang et al., 2019). Also, Ma et al. pointed out DZSM is beneficial for recurrent stroke and NIHSS (Ma et al., 2014). However, Xie found there was no significant difference in the recurrent stroke between the BYHW + WM and WM (Xie, 2015), and we guessed it was caused by a small sample size at that time. Moreover, according to the ranking of SUCRA probabilities, NMT + WM, MXK + WM were most likely to become the best treatments for recurrent stroke, and BYHW + WM and NXT + WM were most probably to be the best choices for NIHSS.

The secondary outcomes were stroke risk factors including Fib, FBG, TG, and TC (Diener and Hankey, 2020). The SCURA ranking of WM alone was very low, implying that the combination of TCM and WM was superior to WM. NST was lower in SUCRA values compared with WM when it comes to TC and TG. We believe that the reason behind NST reducing stroke recurrence was due to other stroke risk factors. Moreover, Chen and Yu showed that MXK can decrease Fib which agrees with our results (Chen and Yu, 2020). Our conclusion was also consistent with Wang et al. who proved that NXT is beneficial for reducing TC and TG (Wang et al., 2019). Furthermore, our results were favored by the outcome of the experiments utilizing TCM to reduce blood lipid and blood glucose in mice (Chen et al., 2018; Li et al., 2018). Based on SUCRA value, MXK + WM and BYHW + WM were most likely to be the best choice for reducing Fib. In terms of FBG, MXK had the highest probability to be the best treatment. In TC, the most effective choices were NXT + WM and TXL + WM. Moreover, NXT + WM and TXL + WM were most likely to be the best in TG. However, the samples of some secondary outcome indicators of TCM were less, thus more RCTs are warranted to test our results.

For all-cause mortality, only DZSM and NXT were found to significantly decrease all-cause mortality, all other comparisons have no statistically significant difference. As regards AEs, there was no significant difference in all contrasts. Thus, this implied that all kinds of TCM + WM were more or less tolerable for patients, however, it may be limited to lack of data. Moreover, the AEs predominantly include gastrointestinal reactions, nausea, and giddiness (Peng and Niu, 2014; Bo et al., 2017; Xu, 2017).

In conclusion, TCM + WM were more effective than WM in the long−term secondary prevention for patients with ischemic stroke, and there was no significant difference in the comparisons of AEs. Regarding recurrent stroke, NIHSS, and all-cause mortality, MXK + WM and NST + WM had relatively good preventive effects on long-term secondary prevention through cluster analysis. The basis for confirming the secondary preventive effect of MXK and NST on cerebral infarction is as follows.

The main component of MXK is *Hirudo nipponic*a Whitman (Haemadipsidae, Hirudo) which is the strongest natural thrombin-specific inhibitor discovered yet (Junren et al., 2021). Hirudin inhibits thrombin activity by directly binding with thrombin and plays an anticoagulant role. Second, hirudin has a strong antiplatelet effect, which can inhibit thrombin-induced platelet thrombin binding, platelet activation as well as aggregation (Garcia et al., 2012; Han et al., 2020). Additionally, in comparison with heparin, hirudin has less bleeding and side effects, and it has no allergic reaction and no toxicity (Johnson, 1994; Markwardt, 1994). Furthermore, clinical trials (e.g., Peng, Zhen, Ge, and Wu) showed that MXK has a significant effect on the secondary prevention of ischemic stroke (Peng and Niu, 2014; Zhen, 2015; Ge et al., 2016; Wu and Xiang, 2018).

*Typha orientalis* C. Presl (Typhaceae, Typhae Pollen), *Paeonia lactiflora* Pall (Paeoniaceae, Paeoniae Radix Rubra), *Curcuma phaeocaulis* Valeton (Zingiberaceae, Curcumae Radix), *Gastrodia elata* Blume (Orchidaceae, Gastrodiae Rhizoma), *Rha ponticum uniflorum* (L.) DC (Compositae, Rhapontici Radix) make up NST, a Chinese medication (Luo et al., 2020). It is also extensively used for increasing blood circulation, eliminating blood stasis, and promoting nerve function recovery (Liu et al., 2014). Moreover, a meta-analysis showed that NST has the effect of increasing blood adiponectin, decreasing neurological deficits, and reducing the area of atherosclerotic plaque (Zhang et al., 2019). Since it has been widely used to treat ischemic stroke, Liu and Huang demonstrated that NST + WM is better than WM in recurrent stroke, and the results are significantly different (Liu and Li, 2012; Huang et al., 2014).

## Innovations and Limitations of the Study

This is for the first time that the secondary prevention effects of various kinds of TCM on ischemic stroke as well as their safety were systematically evaluated. Moreover, this is the pioneer study, ranking seven kinds of TCM based on SUCRA value from different indicators, which furnishes the high-quality basis for clinical practice. Finally, strict inclusion and exclusion criteria were established, and the articles were thereby searched comprehensively.

Nevertheless, the risk of bias in most included articles was unclear and no direct comparison between TCM was found, giving rise to a lack of evidence, but we included 47 articles involving a large sample size and the subgroup analysis showed that the transitivity was good. All included RCTs were conducted in China, therefore more RCTs are needed to see whether the findings of this study applied to patients from other regions. Furthermore, clinical heterogeneity might appear because of the diversity and different courses of treatment of Western medicine. Despite these shortcomings, this is the first comparison of the long-term secondary prevention effects of various TCM on ischemic stroke from multiple indexes and provide some valuable suggestions for clinical practice.

## Conclusion

In summary, our systematical analysis revealed that TCM had positive secondary prevention effects on patients with cerebral infarction, and there was no significant difference in the comparisons of AEs. Additionally, MXK + WM and NST + WM had relatively good preventive effects for recurrent stroke, NIHSS, and all-cause mortality. Nevertheless, on account of some limitations, more high-quality, and double-blinded multicenter RCTs with a larger sample size are needed to test and verify our results in the future.

## Data Availability

The original contributions presented in the study are included in the article/Supplementary Material. Further inquiries can be directed to the corresponding authors.
