# Supplementary material for: Comparison of Traditional Chinese Medicine in the Long-Term Secondary Prevention for Patients with Ischemic Stroke: A Systematical Analysis
Source: Front Pharmacol. 2021 Nov 18;12:722975. doi: 10.3389/fphar.2021.722975 (PMC8637749; doi:10.3389/fphar.2021.722975)
Supplement: Supplementary file 1 [file DataSheet1.zip › supplementary material/supplementary material 2.docx]

| WM | conventional western medicine |
| --- | --- |
| TCM | traditional Chinese medicin |
| NMA | network meta-analysis |
| RCTs | randomized controlled trials |
| NXT | Naoxintong capsule |
| TXL | Tongxinluo capsule |
| BYHW | Buyang Huanwu Decoction |
| NMT | Naomaitai capsule |
| DZSM | Dengzhan Shengmai capsule |
| NST | Naoshuantong capsule |
| MXK | Maixuekang capsule |
| NIHSS | National Institute of Health stroke scale |
| Fib | fibrinogen |
| FBG | fasting blood glucose |
| TG | triglycerides |
| TC | total cholesterol |
| AEs | adverse events |

**The abbreviations used in this manuscript**
