# Supplementary material for: Comparison of Traditional Chinese Medicine in the Long-Term Secondary Prevention for Patients with Ischemic Stroke: A Systematical Analysis
Source: Front Pharmacol. 2021 Nov 18;12:722975. doi: 10.3389/fphar.2021.722975 (PMC8637749; doi:10.3389/fphar.2021.722975)
Supplement: Supplementary file 1 [file DataSheet1.zip › supplementary material/supplementary material 3.docx]

**Specific intervention methods in this systematical analysis**

| Study | Experimental group | Control group |
| --- | --- | --- |
| Sun, 2020 | aspirin(100mg/day)+Naoxintong capsule | aspirin(100mg/day) |
| Shi et al., 2011a | aspirin(100mg/day)+Naoxintong capsule | aspirin(100mg/day) |
| Shi et al., 2011b | aspirin(100mg/day)+atorvastatin(20mg/day)+Naoxintong capsule | aspirin(100mg/day)+atorvastatin(20mg/day) |
| Zang et al., 2011 | aspirin+statins+Naoxintong capsule | aspirin+statins |
| Tian et al., 2010 | aspirin(100mg/day)+Naoxintong capsule | aspirin(100mg/day) |
| Jiang, 2012 | aspirin+statins+Naoxintong capsule | aspirin+statins |
| Tu et al., 2013 | aspirin(100mg/day)+Naoxintong capsule | aspirin(100mg/day) |
| Dang et al., 2018 | aspirin+statins+Naoxintong capsule | aspirin+statins |
| Zhang et al., 2008 | aspirin+antihypertensive drugs+simvastatin+Naoxintong capsule | aspirin+antihypertensive drugs+simvastatin |
| Xu, 2017 | aspirin(100mg/day)+Naoxintong capsule | aspirin(100mg/day) |
| Zhou, 2013 | aspirin+antihypertensive drugs+simvastatin+Naoxintong capsule | aspirin+antihypertensive drugs+simvastatin |
| Meng, 2009 | aspirin+antihypertensive drugs+simvastatin+Naoxintong capsule | aspirin+antihypertensive drugs+simvastatin |
| Wang, 2018 | aspirin(100mg/day)+Naoxintong capsule | aspirin(100mg/day) |
| Zhou, 2018 | antiplatelet drugs+simvastatin+Naoxintong capsule | antiplatelet drugs+simvastatin |
| Chen, 2012 | atorvastatin(20mg/day)+Naoxintong capsule | atorvastatin(20mg/day) |
| Lu et al., 2018 | aspirin(100mg/day)+atorvastatin(20mg/day)+Tongxinluo capsule | aspirin(100mg/day)+atorvastatin(20mg/day) |
| Liu and Cao, 2008 | aspirin(100mg/day)+Tongxinluo capsule | aspirin(100mg/day) |
| Song, 2014 | aspirin(100mg/day)+atorvastatin(10mg/day)+Tongxinluo capsule | aspirin(100mg/day)+atorvastatin(10mg/day) |
| Bo et al. 2017 | aspirin+statins+Tongxinluo capsule | aspirin+statins |
| Zhou, 2014 | aspirin+statins+Tongxinluo capsule | aspirin+statins |
| Yan et al., 2008 | aspirin+statins+Tongxinluo capsule | aspirin+statins |
| Chai, 2015 | aspirin(100mg/day)+atorvastatin(10mg/day)+Tongxinluo capsule | aspirin(100mg/day)+atorvastatin(10mg/day) |
| Xue et al., 2014 | aspirin(100mg/day)+atorvastatin(10mg/day)+Tongxinluo capsule | aspirin(100mg/day)+atorvastatin(10mg/day) |
| Guo et al., 2013 | simvastatin(10mg/day)+Tongxinluo capsule | simvastatin(10mg/day) |
| Jiao et al., 2011 | aspirin+statins+Buyang Huanwu decoction | aspirin+statins |
| Yu, 2013 | aspirin+statins+Buyang Huanwu decoction | aspirin+statins |
| Lin and Li, 2012 | aspirin(75mg/day)+Buyang Huanwu decoction | aspirin(75mg/day) |
| Liu, 2019 | aspirin(100mg/day)+Buyang Huanwu decoction | aspirin(100mg/day) |
| Chen and Cao, 2016 | aspirin(100mg/day)+atorvastatin(10mg/day)+Buyang Huanwu decoction | aspirin(100mg/day)+atorvastatin(10mg/day) |
| Sun, 2008 | aspirin+statins+Naomaitai capsule | aspirin+statins |
| Huang and Huang, 2007 | aspirin+statins+Naomaitai capsule | aspirin+statins |
| Liu and Wang, 2006 | aspirin(100mg/day)+Naomaitai capsule | aspirin(100mg/day) |
| Gan, 2007 | aspirin+Naomaitai capsule | aspirin |
| Li et al., 2014 | aspirin+statins+Naomaitai capsule | aspirin+statins |
| Liu, 2009 | aspirin(75~100mg/day)+antihypertensive drugs+statins+Dengzhan Shenmai capslue | aspirin(75~100mg/day)+antihypertensive drugs+statins |
| Nan and Li, 2016 | aspirin(100mg/day)+Dengzhan Shenmai capslue | aspirin(100mg/day) |
| Chao, 2011 | aspirin(75~150mg/day)+antihypertensive drugs+statins+Dengzhan Shenmai capslue | aspirin(75~150mg/day)+antihypertensive drugs+statins |
| Chen et al., 2008 | aspirin+statins+Dengzhan Shenmai capslue | aspirin+statins |
| Ma et al., 2014 | aspirin(100mg/day)+atorvastatin(20mg/day)+Dengzhan Shenmai capslue | aspirin(100mg/day)+atorvastatin(20mg/day) |
| Liu and Li, 2012 | aspirin+statins+Naoshuantong capsule | aspirin+statins |
| Ye et al., 2015 | aspirin+Naoshuantong capsule | aspirin |
| Huang et al., 2014 | aspirin(100mg/day)+statins+Naoshuantong capsule | aspirin(100mg/day)+statins |
| Peng et al., 2012 | aspirin(100mg/day)+atorvastatin(20mg/day)+Naoshuantong capsule | aspirin(100mg/day)+atorvastatin(20mg/day) |
| Ge et al., 2016 | clopidogrel(75mg/day)+Maixuekang capsule | clopidogrel(75mg/day) |
| Peng and Niu, 2014 | aspirin(100mg/day)+atorvastatin(20mg/day)+Maixuekang capsule | aspirin(100mg/day)+atorvastatin(20mg/day) |
| Zhen, 2015 | aspirin(100mg/day)+atorvastatin(20mg/day)+Maixuekang capsule | aspirin(100mg/day)+atorvastatin(20mg/day) |
| Wu and Xiang, 2018 | aspirin+statins+Maixuekang capsule | aspirin+statins |
