# Supplementary material for: Comparison of Traditional Chinese Medicine in the Long-Term Secondary Prevention for Patients with Ischemic Stroke: A Systematical Analysis
Source: Front Pharmacol. 2021 Nov 18;12:722975. doi: 10.3389/fphar.2021.722975 (PMC8637749; doi:10.3389/fphar.2021.722975)
Supplement: Supplementary file 1 [file DataSheet1.zip › supplementary material/supplementary material 4.docx]

**Indications and chemical analysis of each TCM**

| **Preparations** | **Source** | **compositions** | **Amount of each composition** | **Therapeutic claims in TCM** | **Indications** | **Medication attention** | **Quality control reported? (Y/N)** | **Chemical analysis  reported? (Y/N)** |
| --- | --- | --- | --- | --- | --- | --- | --- | --- |
| Naoxintong capsule | Shanxi Buchang Pharmaceutical Co., Ltd | *Astragalus propinquus* Schischkin [Leguminosae, Astragali Radix] | 66g | Supplementing qi and activating blood circulation, removing blood stasis and dredging collaterals | Qi deficiency and blood stagnation, veins and blood stasis, hemiplegia, limb numbness, crooked mouth and eyes, strong tongue, chest arthralgia, heartache, chest tightness, palpitation and shortness of breath; Cerebral infarction, coronary heart disease, angina pectoris | Pregnant women are prohibited | Y - ZYB2072014021 issued by National Medical Preducts Administration [Detail information can be got from https://www.nmpa.gov.cn/xxgk/ggtg/zhybhpzh/zhybhpzhgg/20140504120001552.html] | Y - HPLC [Detail information can be got from Pharmacopoeia of the People's Republic of China (Part I, finished preparations and single flavor preparations, Naoxintong Jiaonang)] |
|  |  | *Paeonia lactiflora* Pall. [Paeoniaceae, Paeoniae Radix Rubra] | 27g |  |  |  |  |  |
|  |  | *Salvia miltiorrhiza* Bunge [Lamiaceae, Salviae miltiorrhizae Radix et Rhizoma] | 27g |  |  |  |  |  |
|  |  | *Angelica sinensis* (Oliv.) Diels [Apiaceae, Angelicae Sinensis Radix] | 27g |  |  |  |  |  |
|  |  | *Ligusticum striatum* DC. [Apiaceae, Chuanxiong Rhizoma] | 27g |  |  |  |  |  |
|  |  | *Prunus davidiana* (CarriŠre) Franch. [Rosaceae, Persicae Semen] | 27g |  |  |  |  |  |
|  |  | *Carthamus tinctorius* L. [Compositae, Carthami Flos] | 13g |  |  |  |  |  |
|  |  | *Boswellia sacra* Flueck*.* [Burseraceae, Olibanum] | 13g |  |  |  |  |  |
|  |  | *Commiphora myrrha* (Nees) Engl. [Burseraceae, Myrrha] | 13g |  |  |  |  |  |
|  |  | *Spatholobus suberectus* Dunn [Leguminosae, Spatholobi Caulis] | 20g |  |  |  |  |  |
|  |  | *Achyranthes bidentata* Blume [Amaranthaceae, Achyranthis Bidentatae Radix] | 27g |  |  |  |  |  |
|  |  | *Cinnamomum cassia* (L.) J.Presl [Lauraceae, Cinnamomi Ramulus] | 20g |  |  |  |  |  |
|  |  | *Morus alba* L. [Moraceae, Mori Ramulus] | 27g |  |  |  |  |  |
|  |  | *Pheretima aspergillum* (E.Perrier) [Megascolecidae, Pheretima] | 27g |  |  |  |  |  |
|  |  | *Buthus martensii* Karsch [Scorpionidae, Scorpio] | 13g |  |  |  |  |  |
|  |  | *Whitmania pigra* Whitman [Haemadipsidae, Hirudo] | 27g |  |  |  |  |  |
| Tongxinluo capsule | Shijiazhuang Yiling Pharmaceutical Co., Ltd | *Panax ginseng* C. A. Mey. [Araliaceae, Ginseng Radix et Rhizoma] | 360g | Supplementing qi and activating blood circulation, dredging collaterals and relieving pain | Angina pectoris of coronary heart disease belongs to the syndrome of deficiency of heart qi and obstruction of blood stasis collaterals. The symptoms include suffocation, tingling and colic in the chest, immobility, palpitation, spontaneous sweating, shortness of breath and fatigue, purple dark tongue or ecchymosis, astringent pulse or generation | Bleeding diseases, pregnant women and women's menstrual period and yin deficiency and fire excess stroke are prohibited | Y - ZYB20799100 issued by National Medical Preducts Administration [Detail information can be got from https://www.nmpa.gov.cn/xxgk/ggtg/zhybhpzh/zhybhpzhgg/19990510010101955.html] | Y - HPLC-Detail information can be got from Pharmacopoeia of the People's Republic of China (Part I, finished preparations and single flavor preparations, Tongxinluo Jiaonang) |
|  |  | *Hirudo nipponica* Whitman [Haemadipsidae, Hirudo] | 720g |  |  |  |  |  |
|  |  | *Buthus martensii* Karsch [Scorpionidae, Scorpio] | 450g |  |  |  |  |  |
|  |  | *Paeonia lactiflora* Pall. [Paeoniaceae, Paeoniae Radix Rubra] | 330g |  |  |  |  |  |
|  |  | *Cryptotympana pustulata* Fabricius [Cicadellidae, Cicadae Periostracum] | 450g |  |  |  |  |  |
|  |  | *Eupolyphaga sinensis* Walker [Blaberidae, Eupolyphaga Steleophaga] | 450g |  |  |  |  |  |
|  |  | *Scolopendra subspinipes mutilans* L. Koch [Scolopendridae, Scolopendra] | 90g |  |  |  |  |  |
|  |  | *Cinnamomum alatum* Lukman. [Lauraceae, Borneolum] | 90g |  |  |  |  |  |
| Buyang Huanwu decoction | —— | *Astragalus propinquus* Schischkin [Leguminosae, Astragali Radix] | 3g | Invigorate Qi and blood circulation, dispel addiction and dredge collaterals (Zhang,2020; Zhang et al.,2013) | Sequelae of stroke, such as hemiplegia, crooked mouth and eyes, astringent language, salivation, dry stool, frequent urination and enuresis (Zhang,2020; Zhang et al.,2013) | Qi deficiency or yin deficiency and yang hyperactivity (Zhang,2020; Zhang et al.,2013) | N | Y - UPLC-MS/MS -Detail information can be got from “Study on the determination method of 10 bioactive components of Buyang Huanwu decoction against atherosclerosis by UPLC-MS/MS” (Wang et al., 2021) |
|  |  | *Angelica sinensis* (Oliv.) Diels [Apiaceae, Angelicae Sinensis Radix] | 3g |  |  |  |  |  |
|  |  | *Paeonia lactiflora* Pall. [Paeoniaceae, Paeoniae Radix Rubra] | 3g |  |  |  |  |  |
|  |  | *Pheretima vulgaris* Chen [Megascolecidae, Pheretima] | 3g |  |  |  |  |  |
|  |  | *Ligusticum striatum* DC. [Apiaceae, Chuanxiong Rhizoma] | 3g |  |  |  |  |  |
|  |  | *Carthamus tinctorius* L. [Compositae, Carthami Flos] | 6g |  |  |  |  |  |
|  |  | *Prunus persica* (L.) Batsch [Rosaceae, Persicae Semen] | 120g |  |  |  |  |  |
| Naomaitai capsule | Guilin Sanjin Pharmaceutical Co., Ltd | *Panax ginseng* C.A.Mey. [Araliaceae, Ginseng Radix et Rhizoma Rubra] | 155g | Supplementing qi and activating blood circulation, quenching wind and eliminating phlegm | Apoplexy with Qi deficiency and blood stasis, wind phlegm and blood stasis blocking the vein syndrome. The symptoms include hemiplegia, crooked tongue, astringent speech, dizziness, half body numbness, shortness of breath and fatigue, and mild symptoms in the recovery stage and acute stage of ischemic stroke | Pregnant women are prohibited | Y - ZYB20720100310 issued by National Medical Preducts Administration [Detail information can be got from https://www.nmpa.gov.cn/xxgk/ggtg/zhybhpzh/zhybhpzhgg/20101104164101660.html] | Y - HPLC-Detail information can be got from Pharmacopoeia of the People's Republic of China (Part I, finished preparations and single flavor preparations, Naomaitai Jiaonang) |
|  |  | *Panax notoginseng* (Burkill) F.H.Chen [Araliaceae, Notoginseng Radix et Rhizoma] | 180g |  |  |  |  |  |
|  |  | Angelica sinensis (Oliv.) Diels [Apiaceae, Angelicae Sinensis Radix] | 120g |  |  |  |  |  |
|  |  | *Salvia miltiorrhiza* Bge. [Lamiaceae, Salviae Miltiorrhizae Radix ET Rhizoma] | 165g |  |  |  |  |  |
|  |  | *Spatholobus suberectus* Dunn [Leguminosae, Spatholobi Caulis] | 150g |  |  |  |  |  |
|  |  | *Carthamus tinctorius* L. [Compositae, Carthami Flos] | 120g |  |  |  |  |  |
|  |  | *Ginkgo biloba* L. [Ginkgoaceae, Ginkgo Folium] | 180g |  |  |  |  |  |
|  |  | *Crataegus pinnatifida* Bunge [Rosaceae, Crataegi Fructus] | 150g |  |  |  |  |  |
|  |  | *Chrysanthemum morifolium* Ramat. [Compositae, Chrysanthemi Flos] | 120g |  |  |  |  |  |
|  |  | *Haliotis diversicolor* Reeve [Haliotidae, Haliotidis Concha] | 120g |  |  |  |  |  |
|  |  | *Reynoutria multiflora* (Thunb.) Moldenke [Polygonaceae, Polygoni Multiflori Radix] | 150g |  |  |  |  |  |
|  |  | *Acorus calamus var. angustatus* Besser [Acoraceae, Acori Tatarinowii Rhizoma] | 105g |  |  |  |  |  |
|  |  | *Pueraria montana* var. *lobata* (Willd.) Sanjappa & Pradeep [Leguminosae, Puerariae Lobatae Radix] | 150g |  |  |  |  |  |
| Dengzhan Shengmai capslue | Yunnan BioValley Pharmaceutical Co., Ltd | *Erigeron breviscapus* (Vaniot) Hand.-Mazz. [Compositae, Erigerontis Herba] | 3000g | Supplementing qi and nourishing Yin, promoting blood circulation and brain | Chest arthralgia and heartache caused by deficiency of Qi and Yin, blood stasis blocking brain collaterals, sequelae of stroke, dementia, amnesia and numbness of hands and feet; Coronary heart disease, angina pectoris, ischemic cardiovascular and cerebrovascular disease, hyperlipidemia | It is forbidden in acute stage of intracerebral hemorrhage | N | Y - HPLC-Detail information can be got from Pharmacopoeia of the People's Republic of China (Part I, finished preparations and single flavor preparations, Dengzhan Shengmai Jiaonang) |
|  |  | *Panax ginseng* C. A. Mey. [Araliaceae, Ginseng Radix et Rhizoma] | 600g |  |  |  |  |  |
|  |  | *Schisandra chinensis* (Turcz.) Baill. [Schisandraceae, Schisandrae Chinensis Fructus] | 600g |  |  |  |  |  |
|  |  | *Ophiopogon japonicus* (Thunb.) Ker Gawl. [Asparagaceae, Ophiopogonis Radix] | 1100g |  |  |  |  |  |
| Naoshuantong capsule | Guangdong South China Pharmaceutical Group Co., Ltd | *Typha orientalis* C.Presl [Typhaceae, Typhae Pollen] | 890g | Promoting blood circulation and dredging collaterals, dispelling wind and resolving phlegm | In the ischemic stroke caused by wind phlegm and blood stasis blocking veins, in the acute and convalescent stages of meridians, hemiplegia, crooked tongue, adverse language or aphasia, partial numbness, shortness of breath, fatigue or dizziness, tinnitus, dull or dark red tongue, thin white or greasy fur, thin pulse or thin string, smooth string | (1) A few patients may have stomach and wrist noise, discomfort, constipation, etc. (2) Use with caution. (3) Forbidden for pregnant women. | N | Y - HPLC-Detail information can be got from Pharmacopoeia of the People's Republic of China (Part I, finished preparations and single flavor preparations,Naoshuantong Jiaonang) |
|  |  | *Paeonia lactiflora* Pall. [Paeoniaceae, Paeoniae Radix Rubra] | 635g |  |  |  |  |  |
|  |  | *Curcuma phaeocaulis* Valeton [Zingiberaceae, Curcumae Radix] | 510g |  |  |  |  |  |
|  |  | *Gastrodia elata* Blume [Orchidaceae, Gastrodiae Rhizoma] | 255g |  |  |  |  |  |
|  |  | *Rha ponticum uniflorum* (L.) DC. [Compositae, Rhapontici Radix] | 380g |  |  |  |  |  |
| Maixuekang capsule | Chongqing duoputai Pharmaceutical Co., Ltd | *Hirudo nipponic*a Whitman [Haemadipsidae, Hirudo] | 0.25g | Breaking blood, removing blood stasis and dredging pulse (Qin and Zhang, 2003) | Blood stasis, amenorrhea, leprosy, apoplexy, hemiplegia, traumatic injury (Qin and Zhang, 2003) | Patients with allergic constitution and severe digestive system should use drugs carefully (Zhou et al., 2003) | Y - ZYB20720130110 issued by National Medical Preducts Administration [Detail information can be got from https://www.nmpa.gov.cn/xxgk/ggtg/zhybhpzh/zhybhpzhgg/20130903120001476.html] | N |

**Reference:**

Committee of National Pharmacopoeia. (2000). *Pharmacopoeia of the People's Republic of China*. Chemical Industry Press.

Huang, W., and Zhang, B., Hua (2011). Study on the application of Maixuekang capsule. *Capital Medicine* (20), 30-32. doi: 10.3969/j.issn.1005-8257.2011.20.019.

Qin, J.L., and Zhang, W.H. (2003). Study on anti hypoxia effect of Naoshuantong. *Chinese Archives of Traditional Chinese Medicine* 21(7), 1100-1100. doi: 10.3969/j.issn.1673-7717.2003.07.036.

Wang, W.W., Jiang, L., Zhang, Q.Y., Li, B.T., Zeng, Z.J., Liu, Y.H., et al. (2021). Study on the determination method of 8 bioactive components of Buyang Huanwu decoction against atherosclerosis by UPLC-MS/MS. *Chinese Journal of Pharmaceutical Analysis* 41(01), 29-41. doi: 10.16155/j.0254-1793.2021.01.04.

Zhang, W.J. (2020). Clinical examples of Buyang Huanwu Decoction. *China's Naturopathy* 28(15), 103-104. doi: 10.19621/j.cnki.11-3555/r.2020.1546.

Zhou, J.M., Pan, Z.J., and wang, S.L. (2003). Pharmacodynamic and Toxicologic Research on Nao Mai Kang Capsule. *China Journal of Traditional Chinese Medicine and Pharmacy* 18(7), 408-413. doi: 10.3969/j.issn.1673-1727.2003.07.008.

Zhang, C., Duan, X.Y., and Yang, Z.W. (2013). Clinical Application of Buyang Huanwu Decoction. *Chin. Gen. Pract.* 16(16), 1918-1919,1926. doi: 10.3969/j.issn.1007-9572.2013.06.031.
