# Supplementary material for: Comparison of Traditional Chinese Medicine in the Long-Term Secondary Prevention for Patients with Ischemic Stroke: A Systematical Analysis
Source: Front Pharmacol. 2021 Nov 18;12:722975. doi: 10.3389/fphar.2021.722975 (PMC8637749; doi:10.3389/fphar.2021.722975)
Supplement: Supplementary file 1 [file DataSheet1.zip › supplementary material/supplementary material 5.docx]

**Compositions and extraction procedure of each TCM**

| **Preparations** | **compositions** | **Amount of each composition** | **Extraction procedure (compositions)** | **Extraction procedure (preparations)** | **Quality control reported? (Y/N)** | **Chemical analysis  reported? (Y/N)** |
| --- | --- | --- | --- | --- | --- | --- |
| Naoxintong capsule | *Astragalus propinquus* Schischkin [Leguminosae, Astragali Radix] | 66g | Remove impurities, separate sizes, wash, moisten thoroughly, cut into thick pieces and dry | Take *Pheretima aspergillum* (E.Perrier) [Megascolecidae, Pheretima] and *Buthus martensii* Karsch [Scorpionidae, Scorpio] and crush them into fine powder; The rest 14 flavors are crushed into fine powder, grinded with the above powder, screened, mixed, put into capsules, and made into 1000 capsules. | Y - ZYB2072014021 issued by National Medical Preducts Administration [Detail information can be got from https://www.nmpa.gov.cn/xxgk/ggtg/zhybhpzh/zhybhpzhgg/20140504120001552.html] | Y - HPLC [Detail information can be got from Pharmacopoeia of the People's Republic of China (Part I, finished preparations and single flavor preparations, Naoxintong Jiaonang)] |
|  | *Paeonia lactiflora* Pall. [Paeoniaceae, Paeoniae Radix Rubra] | 27g | Remove impurities, separate size, wash, moisten thoroughly, cut thick pieces and dry |  |  |  |
|  | *Salvia miltiorrhiza* Bunge [Lamiaceae, Salviae miltiorrhizae Radix et Rhizoma] | 27g | Remove impurities and residual stems, wash, moisten thoroughly, cut thick slices and dry |  |  |  |
|  | *Angelica sinensis* (Oliv.) Diels [Apiaceae, Angelicae Sinensis Radix] | 27g | Remove impurities, wash, moisten, slice, dry in the sun or at low temperature |  |  |  |
|  | *Ligusticum striatum* DC. [Apiaceae, Chuanxiong Rhizoma] | 27g | Remove impurities, separate size, wash, moisten thoroughly, cut thick pieces and dry |  |  |  |
|  | *Prunus davidiana* (CarriŠre) Franch. [Rosaceae, Persicae Semen] | 27g | Remove impurities. Mash when needed |  |  |  |
|  | *Carthamus tinctorius* L. [Compositae, Carthami Flos] | 13g | Remove impurities |  |  |  |
|  | *Boswellia sacra* Flueck*.* [Burseraceae, Olibanum] | 13g | Take the pure frankincense and fry it with vinegar (general rule 0213) until the surface is bright.5kg vinegar per 100kg Boswellia sacra Flueck. [Burseraceae, Olibanum] |  |  |  |
|  | *Commiphora myrrha* (Nees) Engl. [Burseraceae, Myrrha] | 13g | Take the net myrrh and fry it with vinegar (general rule 0213) until the surface is bright. 5kg of vinegar per 100kg Commiphora myrrha (Nees) Engl. [Burseraceae, Myrrha] |  |  |  |
|  | *Spatholobus suberectus* Dunn [Leguminosae, Spatholobi Caulis] | 20g | Remove branches and leaves, slice and dry |  |  |  |
|  | *Achyranthes bidentata* Blume [Amaranthaceae, Achyranthis Bidentatae Radix] | 27g | Remove impurities, wash, moisten thoroughly, remove residual reed heads, cut into sections and dry |  |  |  |
|  | *Cinnamomum cassia* (L.) J.Presl [Lauraceae, Cinnamomi Ramulus] | 20g | Remove impurities, wash, moisten thoroughly, cut into thick pieces and dry |  |  |  |
|  | *Morus alba* L. [Moraceae, Mori Ramulus] | 27g | If not sliced, wash, moisten thoroughly, cut into thick pieces and dry |  |  |  |
|  | *Pheretima aspergillum* (E.Perrier) [Megascolecidae, Pheretima] | 27g | Remove impurities, wash, cut into sections and dry |  |  |  |
|  | *Buthus martensii* Karsch [Scorpionidae, Scorpio] | 13g | Remove impurities, wash and dry |  |  |  |
|  | *Whitmania pigra* Whitman [Haemadipsidae, Hirudo] | 27g | Wash, cut and dry |  |  |  |
| Tongxinluo capsule | *Panax ginseng* C. A. Mey. [Araliaceae, Ginseng Radix et Rhizoma] | 360g | Moisten thoroughly, slice, dry, or crush and mash when used | Select eight herbs respectively, and grind 90g Cinnamomum alatum Lukman. [Lauraceae, Borneolum] separately; Wash *Hirudo nipponica* Whitman [Haemadipsidae, Hirudo], *Buthus martensii* Karsch [Scorpionidae, Scorpio], *Cryptotympana pustulata* Fabricius [Cicadellidae, Cicadae Periostracum], *Eupolyphaga sinensis* Walker [Blaberidae, Eupolyphaga Steleophaga], *Scolopendra subspinipe*s mutilans L. Koch [Scolopendridae, Scolopendra]. Remove them and put them into the oven, then dry at low temperature, take 720 grams of *Hirudo nipponica* Whitman [Haemadipsidae, Hirudo], 450 grams of *Buthus martensii* Karsch [Scorpionidae, Scorpio], 450 grams of *Cryptotympana pustulata* Fabricius [Cicadellidae, Cicadae Periostracum], 90 grams of *Eupolyphaga sinensis* Walker [Blaberidae, Eupolyphaga Steleophaga], 450 grams of *Scolopendra subspinipes mutilans* L. Koch [Scolopendridae, Scolopendra], combine them together and crush them into fine powder (120 mesh). 360 g *Panax ginseng* C. A. Mey. [Araliaceae, Ginseng Radix et Rhizoma] was refluxed and extracted twice with 2400ml (about 2160 g) ethanol solution with a concentration of 70%. The first time was 3 hours and the second time was 2 hours. The extract was combined, and the ethanol was recovered until there was no alcohol taste. The ginseng residue was used for standby; Add 330 grams of *Paeonia lactiflora* Pall. [Paeoniaceae, Paeoniae Radix Rubra] into the residue and aqueous solution of ginseng, add 26460 grams of water, heat and decoct twice, the first time for 3 hours, the second time for 2 hours, combine the decoctions, concentrate to a clear paste with a relative excess of 1.23 g / cm3 (measured at 60 ℃), then add the ginseng alcohol extract, mix well, put it into the oven, dry at 65 ℃ and crush it into fine powder; Grind *Cinnamomum alatum* Lukman. [Lauraceae, Borneolum] and the above animal drugs evenly, then mix it with *Paeonia lactiflora* Pall. [Paeoniaceae, Paeoniae Radix Rubra] and other extract powder, put it into the capsule, polish the capsule and pack it into a box. Each capsule contains 0.38g crude medicine (Wu, 1997). | Y - ZYB20799100 issued by National Medical Preducts Administration [Detail information can be got from https://www.nmpa.gov.cn/xxgk/ggtg/zhybhpzh/zhybhpzhgg/19990510010101955.html] | Y - HPLC-Detail information can be got from Pharmacopoeia of the People's Republic of China (Part I, finished preparations and single flavor preparations, Tongxinluo Jiaonang) |
|  | *Hirudo nipponica* Whitman [Haemadipsidae, Hirudo] | 720g | Wash, cut and dry |  |  |  |
|  | *Buthus martensii* Karsch [Scorpionidae, Scorpio] | 450g | Remove impurities, wash and dry |  |  |  |
|  | *Paeonia lactiflora* Pall. [Paeoniaceae, Paeoniae Radix Rubra] | 330g | Remove impurities, separate size, wash, moisten thoroughly, cut thick pieces and dry |  |  |  |
|  | *Cryptotympana pustulata* Fabricius [Cicadellidae, Cicadae Periostracum] | 450g | Remove impurities, wash and dry |  |  |  |
|  | *Eupolyphaga sinensis* Walker [Blaberidae, Eupolyphaga Steleophaga] | 450g | Scald in boiling water, dry or dry |  |  |  |
|  | *Scolopendra subspinipes mutilans* L. Koch [Scolopendridae, Scolopendra] | 90g | Remove bamboo slices, wash, bake yellow over low heat, and cut into sections |  |  |  |
|  | *Cinnamomum alatum* Lukman. [Lauraceae, Borneolum] | 90g | Fresh branches and leaves are extracted and processed |  |  |  |
| Buyang Huanwu decoction | *Astragalus propinquus* Schischkin [Leguminosae, Astragali Radix] | 3g | Remove impurities, separate sizes, wash, moisten thoroughly, cut into thick pieces and dry | Soak the decoction pieces in local warm water for 30 minutes and decoct them for 3 times. Add 8 times of water for the first time, 6 times of water for the second time and 4 times of water for the third time. Combine the filtrates of the three times of decocting and polymerize them until the final mass concentration of crude medicine is 1.26 g / ml (Liu, 2019). | N | Y - UPLC-MS/MS -Detail information can be got from “Study on the determination method of 10 bioactive components of Buyang Huanwu decoction against atherosclerosis by UPLC-MS/MS” (Wang et al., 2021). |
|  | *Angelica sinensis* (Oliv.) Diels [Apiaceae, Angelicae Sinensis Radix] | 3g | Remove impurities, wash, moisten, slice, dry in the sun or at low temperature |  |  |  |
|  | *Paeonia lactiflora* Pall. [Paeoniaceae, Paeoniae Radix Rubra] | 3g | Remove impurities, separate size, wash, moisten thoroughly, cut thick pieces and dry |  |  |  |
|  | *Pheretima vulgaris* Chen [Megascolecidae, Pheretima] | 3g | Remove impurities, wash, cut into sections and dry |  |  |  |
|  | *Ligusticum striatum* DC. [Apiaceae, Chuanxiong Rhizoma] | 3g | Remove impurities, separate size, wash, moisten thoroughly, cut thick pieces and dry |  |  |  |
|  | *Carthamus tinctorius* L. [Compositae, Carthami Flos] | 6g | Remove impurities |  |  |  |
|  | *Prunus persica* (L.) Batsch [Rosaceae, Persicae Semen] | 120g | Remove impurities. Mash when needed |  |  |  |
| Naomaitai capsule | *Panax ginseng* C.A.Mey. [Araliaceae, Ginseng Radix et Rhizoma Rubra] | 155g | Moisten thoroughly, slice, dry, crush or mash when used | For the above thirteen flavors, 50g of *Panax ginseng* C.A.Mey. [Araliaceae, Ginseng Radix et Rhizoma Rubra] and *Panax notoginseng* (Burkill) F.H.Chen [Araliaceae, Notoginseng Radix et Rhizoma] are crushed into fine powder. The remaining *Panax ginseng* C.A.Mey. [Araliaceae, Ginseng Radix et Rhizoma Rubra], *Panax notoginseng* (Burkill) F.H.Chen [Araliaceae, Notoginseng Radix et Rhizoma], *Salvia miltiorrhiza* Bge. [Lamiaceae, Salviae Miltiorrhizae Radix ET Rhizoma] and *Ginkgo biloba* L. [Ginkgoaceae, Ginkgo Folium] are heated and refluxed with 60% ethanol for 2 hours, filtered, and the ethanol is recovered from the filtrate. The drug residue and other nine flavors such as *Angelica sinensis* (Oliv.) Diels [Apiaceae, Angelicae Sinensis Radix] are decocted twice with water for 2 hours each time. The decoction is combined, filtered, the filtrate is combined with alcohol extract, concentrated to a clear paste with a relative density of 1.24 ~ 1.26 (60 ℃), dried, crushed, added with the above fine powder and an appropriate amount of auxiliary materials, mixed, granulated, loaded into capsules, and made into 1000 capsules. | Y - ZYB20720100310 issued by National Medical Preducts Administration [Detail information can be got from https://www.nmpa.gov.cn/xxgk/ggtg/zhybhpzh/zhybhpzhgg/20101104164101660.html] | Y - HPLC-Detail information can be got from Pharmacopoeia of the People's Republic of China (Part I, finished preparations and single flavor preparations, Naomaitai Jiaonang) |
|  | *Panax notoginseng* (Burkill) F.H.Chen [Araliaceae, Notoginseng Radix et Rhizoma] | 180g | Take Panax notoginseng, wash, dry and grind into fine powder |  |  |  |
|  | Angelica sinensis (Oliv.) Diels [Apiaceae, Angelicae Sinensis Radix] | 120g | Remove impurities, wash, moisten, slice, dry in the sun or at low temperature |  |  |  |
|  | *Salvia miltiorrhiza* Bge. [Lamiaceae, Salviae Miltiorrhizae Radix ET Rhizoma] | 165g | Remove impurities and residual stems, wash, moisten thoroughly, cut thick slices and dry |  |  |  |
|  | *Spatholobus suberectus* Dunn [Leguminosae, Spatholobi Caulis] | 150g | Remove branches and leaves, slice and dry |  |  |  |
|  | *Carthamus tinctorius* L. [Compositae, Carthami Flos] | 120g | Remove impurities |  |  |  |
|  | *Ginkgo biloba* L. [Ginkgoaceae, Ginkgo Folium] | 180g | Harvest the leaves when they are still green in autumn and dry them in time |  |  |  |
|  | *Crataegus pinnatifida* Bunge [Rosaceae, Crataegi Fructus] | 150g | Remove impurities and exfoliated nuclei |  |  |  |
|  | *Chrysanthemum morifolium* Ramat. [Compositae, Chrysanthemi Flos] | 120g | Dry in the shade or bake, or smoke, steam and dry in the sun |  |  |  |
|  | *Haliotis diversicolor* Reeve [Haliotidae, Haliotidis Concha] | 120g | Remove impurities, wash, dry and crush |  |  |  |
|  | *Reynoutria multiflora* (Thunb.) Moldenke [Polygonaceae, Polygoni Multiflori Radix] | 150g | Remove impurities, wash, soak slightly, moisten thoroughly, cut into thick pieces or blocks, and dry |  |  |  |
|  | *Acorus calamus var. angustatus* Besser [Acoraceae, Acori Tatarinowii Rhizoma] | 105g | Remove impurities, wash, moisten thoroughly, cut into thick pieces and dry |  |  |  |
|  | *Pueraria montana* var. *lobata* (Willd.) Sanjappa & Pradeep [Leguminosae, Puerariae Lobatae Radix] | 150g | Remove impurities, wash, moisten thoroughly, cut into thick pieces and dry in the sun |  |  |  |
| Dengzhan Shengmai capslue | *Erigeron breviscapus* (Vaniot) Hand.-Mazz. [Compositae, Erigerontis Herba] | 3000g | Remove impurities and dry in the sun. | For the above four flavors, take *Erigeron breviscapus* (Vaniot) Hand.-Mazz. [Compositae, Erigerontis Herba], add 80% ~ 90% ethanol for reflux extraction for three times, filter, combine the filtrate, and concentrate into extract under reduced pressure; Add three times the amount of water to dissolve the extract, add 10% sodium hydroxide to facilitate dissolution under stirring, adjust the pH value to 8, filter, add 10% sulfuric acid to adjust the pH value to 3, place for 2 hours, filter, collect precipitation, wash with water to neutral, and standby. The remaining three flavors, plus 80% to 90% ethanol reflux for three times, filtered, combined with filtrate, decompressed and concentrated, extracted with n-butanol for three times, combined with extract, decompressed and recovered butanol and condensed to thick paste, thickened paste combined with the above precipitates, adding 2-fold amount of water to dissolve, adding dilute sodium hydroxide to adjust pH to 7, filtrating, filtrating spray drying, adding starch and magnesium stearate. Mix well, put into capsules and make 1000 capsules. | N | Y - HPLC-Detail information can be got from Pharmacopoeia of the People's Republic of China (Part I, finished preparations and single flavor preparations, Dengzhan Shengmai Jiaonang) |
|  | *Panax ginseng* C. A. Mey. [Araliaceae, Ginseng Radix et Rhizoma] | 600g | Moisten thoroughly, slice, dry, or crush and mash when used. |  |  |  |
|  | *Schisandra chinensis* (Turcz.) Baill. [Schisandraceae, Schisandrae Chinensis Fructus] | 600g | Remove impurities. Mash when needed. |  |  |  |
|  | *Ophiopogon japonicus* (Thunb.) Ker Gawl. [Asparagaceae, Ophiopogonis Radix] | 1100g | Remove impurities, wash, moisten, flatten and dry. |  |  |  |
| Naoshuantong capsule | *Typha orientalis* C.Presl [Typhaceae, Typhae Pollen] | 890g | Knead the lumps and sift them | For the above five flavors, *Paeonia lactiflora* Pall. [Paeoniaceae, Paeoniae Radix Rubra] is heated and refluxed with 70% ethanol for extraction twice, 1 hour each time. The extract is combined, filtered, the filtrate is recovered with ethanol and concentrated to an appropriate amount, dried, crushed, added with an appropriate amount of calcium hydrogen phosphate, mixed, and dry paste powder for standby; *Curcuma phaeocaulis* Valeton [Zingiberaceae, Curcumae Radix] is heated and refluxed with 80% ethanol for extraction twice, 1 hour each time. The extract is combined, filtered and the filtrate is used for standby; The drug residue, *Typha orientalis* C.Presl [Typhaceae, Typhae Pollen], *Gastrodia elata* Blume [Orchidaceae, Gastrodiae Rhizoma] and *Rha ponticum uniflorum* (L.) DC. [Compositae, Rhapontici Radix] are decocted twice with water for 1 hour each time. The decoctions are combined, filtered, the filtrate is concentrated to the clear paste with relative density of 1.04 ~ 1.10 (40 ℃), the alcohol content is 70% with ethanol, refrigerated for 48 hours, the supernatant is combined with the alcohol extract of Curcuma, the ethanol is recovered, concentrated to an appropriate amount, dried, crushed, and an appropriate amount of calcium hydrogen phosphate is added, Combine with red peony dry cream powder, granulate with hydroxypropyl methylcellulose ethanol solution, dry, add an appropriate amount of talc powder, silica and magnesium stearate, mix well, and put into capsules to make 1000 tablets. | N | Y - HPLC-Detail information can be got from Pharmacopoeia of the People's Republic of China (Part I, finished preparations and single flavor preparations,Naoshuantong Jiaonang) |
|  | *Paeonia lactiflora* Pall. [Paeoniaceae, Paeoniae Radix Rubra] | 635g | Remove impurities, separate size, wash, moisten thoroughly, cut thick pieces and dry. |  |  |  |
|  | *Curcuma phaeocaulis* Valeton [Zingiberaceae, Curcumae Radix] | 510g | Wash, moisten, slice and dry |  |  |  |
|  | *Gastrodia elata* Blume [Orchidaceae, Gastrodiae Rhizoma] | 255g | Wash, moisten or steam soft, slice and dry |  |  |  |
|  | *Rha ponticum uniflorum* (L.) DC. [Compositae, Rhapontici Radix] | 380g | Remove impurities, wash, moisten thoroughly, cut into thick pieces and dry in the sun |  |  |  |
| Maixuekang capsule | *Hirudo nipponic*a Whitman [Haemadipsidae, Hirudo] | 0.25g | Wash, cut and dry | Freezing and low temperature drying (Song et al., 2018) | Y - ZYB20720130110 issued by National Medical Preducts Administration [Detail information can be got from https://www.nmpa.gov.cn/xxgk/ggtg/zhybhpzh/zhybhpzhgg/20130903120001476.html] | N |

**Reference:**

Committee of National Pharmacopoeia. (2000). *Pharmacopoeia of the People's Republic of China*. Chemical Industry Press.

Liu, F. (2019). Treatment of 24 cases of ischemic stroke with Buyang Huanwu Decoction Combined with aspirin. *China Practical Medicine* 14(3), 119-120. doi: 10.14163/j.cnki.11-5547/r.2019.03.072.

Song, J., Lyu, Y., Wang, M., Zhang, J., Gao, L., and Tong, X. (2018). Treatment of Human Urinary Kallidinogenase Combined with Maixuekang Capsule Promotes Good Functional Outcome in Ischemic Stroke. *Front Physiol* 9, 84. doi: 10.3389/fphys.2018.00084.

Wang, W.W., Jiang, L., Zhang, Q.Y., Li, B.T., Zeng, Z.J., Liu, Y.H., et al. (2021). Study on the determination method of 8 bioactive components of Buyang Huanwu decoction against atherosclerosis by UPLC-MS/MS. *Chinese Journal of Pharmaceutical Analysis* 41(01), 29-41. doi: 10.16155/j.0254-1793.2021.01.04.

Wu, Y. L. (1997). Tongxinluo capsule for treating coronary heart disease and angina pectoris and its preparation method. CN, CN1198332 A.
